# The impact of PPARα activation on whole genome gene expression in human precision cut liver slices

**DOI:** 10.1186/s12864-015-1969-3

**Published:** 2015-10-08

**Authors:** Aafke W.F. Janssen, Bark Betzel, Geert Stoopen, Frits J. Berends, Ignace M. Janssen, Ad A. Peijnenburg, Sander Kersten

**Affiliations:** Nutrition, Metabolism and Genomics Group, Wageningen University, Stippeneng 4, 6708 WE, Wageningen, The Netherlands; Department of Surgery, Rijnstate Hospital, Wagnerlaan 55, 6815 AD, Arnhem, The Netherlands; RIKILT—Institute of Food Safety, Wageningen UR, P.O. Box 230, 6700 AE Wageningen, The Netherlands

**Keywords:** Precision cut liver slices, PPARα, Human liver, Transcriptomics

## Abstract

**Background:**

Studies in mice have shown that PPARα is an important regulator of lipid metabolism in liver and key transcription factor involved in the adaptive response to fasting. However, much less is known about the role of PPARα in human liver.

**Methods:**

Here we set out to study the function of PPARα in human liver via analysis of whole genome gene regulation in human liver slices treated with the PPARα agonist Wy14643.

**Results:**

Quantitative PCR indicated that PPARα is well expressed in human liver and human liver slices and that the classical PPARα targets PLIN2, VLDLR, ANGPTL4, CPT1A and PDK4 are robustly induced by PPARα activation. Transcriptomics analysis indicated that 617 genes were upregulated and 665 genes were downregulated by PPARα activation (*q* value < 0.05). Many genes induced by PPARα activation were involved in lipid metabolism (ACSL5, AGPAT9, FADS1, SLC27A4), xenobiotic metabolism (POR, ABCC2, CYP3A5) or the unfolded protein response, whereas most of the downregulated genes were involved in immune-related pathways. Among the most highly repressed genes upon PPARα activation were several chemokines (e.g. CXCL9-11, CCL8, CX3CL1, CXCL6), interferon γ-induced genes (e.g. IFITM1, IFIT1, IFIT2, IFIT3) and numerous other immune-related genes (e.g. TLR3, NOS2, and LCN2). Comparative analysis of gene regulation by Wy14643 between human liver slices and primary human hepatocytes showed that down-regulation of gene expression by PPARα is much better captured by liver slices as compared to primary hepatocytes. In particular, PPARα activation markedly suppressed immunity/inflammation-related genes in human liver slices but not in primary hepatocytes. Finally, several putative new target genes of PPARα were identified that were commonly induced by PPARα activation in the two human liver model systems, including TSKU, RHOF, CA12 and VSIG10L.

**Conclusion:**

Our paper demonstrates the suitability and superiority of human liver slices over primary hepatocytes for studying the functional role of PPARα in human liver. Our data underscore the major role of PPARα in regulation of hepatic lipid and xenobiotic metabolism in human liver and reveal a marked immuno-suppressive/anti-inflammatory effect of PPARα in human liver slices that may be therapeutically relevant for non-alcoholic fatty liver disease.

**Electronic supplementary material:**

The online version of this article (doi:10.1186/s12864-015-1969-3) contains supplementary material, which is available to authorized users.

## Background

The Peroxisome Proliferator Activated Receptors (PPARs) represent an important group of receptors involved in mediating the pleiotropic effects of various environmental contaminants, food components, and drugs [1]. PPARs are members of the nuclear receptor superfamily and induce the expression of numerous genes by functioning as ligand-activated transcription factors. The ligands for PPARs encompass a range of synthetic compounds and endogenous lipids, including various fatty acids and eicosanoids. Three different PPAR subtypes can be distinguished: PPARα, PPARδ, and PPARγ, each characterized by a distinct tissue expression profile and set of functions [2, 3]. Multiple functions have been assigned to PPARδ, including roles in inflammation, lipid metabolism and cancer [4]. Due to its ubiquitous expression pattern and diverse cellular actions, no single descriptor appropriately captures the biological function of PPARδ. The PPARγ is known as the key transcriptional regulator that drives adipogenesis [5], the process by which fat cells differentiate from pre-adipocytes into mature adipose cells. Apart from adipocytes, PPARγ is also expressed in a limited number of other cell types where it exerts anti-inflammatory actions and promotes lipid storage [6]. By serving as the molecular target of the insulin-sensitizing drugs pioglitazone and rosiglitazone, PPARγ is one of the key receptors in the pharmacological treatment of type 2 diabetes [7].

PPARα is best known for its role in the liver, where it acts as the master regulator of lipid metabolism, especially during fasting [8–10]. Fasting is associated with dramatic changes in lipid handling in the liver, which is coordinated by PPARα. Specifically, low and high throughput gene expression analyses have demonstrated that PPARα governs expression of numerous genes involved in nearly every single aspect of lipid metabolism, including fatty acid uptake, mitochondrial and peroxisomal fatty acid oxidation, ketogenesis, and formation and breakdown of triglycerides and lipid droplets [11].

Similar to other members of the PPAR family, PPARα is activated by a range of different fatty acids and eicosanoids [12–16]. In addition, PPARα serves as receptor for a diverse array of synthetic compounds collectively referred to as peroxisome proliferators [17]. These include phthalates, insecticides, herbicides, surfactants, organic solvents, and hypolipidemic fibrate drugs. Fibrates have been used for several decades mainly for their ability to lower circulating triglycerides [18]. More recently, pharmacological targeting of PPARα has shown promise for the treatment of non-alcoholic fatty liver disease. Specifically, the dual PPARα/δ agonist GFT505 was shown to improve liver dysfunction markers, decrease hepatic lipid accumulation, and reduce inflammatory gene expression in liver in several animal models of non-alcoholic fatty liver disease (NAFLD) [19]. Furthermore, GFT505 treatment lowered liver dysfunction markers and improved hepatic and peripheral insulin sensitivity in human subjects [19, 20].

Most of our insights into the physiological, toxicological and pharmacological role of PPARα has been derived from experiments in animals and in particular from rodent studies. These studies have revealed that PPARα is not only involved in the adaptive response to fasting but also mediates the hepatocarcinogenic effects of peroxisome proliferators [21]. Whether PPARα exerts similar functions in human liver has been the subject of controversy [22], which has been fueled by the perceived lack of effect of PPARα agonists on peroxisomal fatty acid oxidation in humans [23], as well as due to the presumed low expression of PPARα in human liver [24]. However, more recent studies have partly refuted those notions, showing that a) PPARα expression is similar in mouse and human liver, b) in human hepatocytes PPARα governs the expression of numerous genes in various lipid metabolic pathways, including peroxisomal fatty acid oxidation [25, 26].

Nevertheless, the absence of more complex human model systems has hampered our ability to gain insight into the molecular function of PPARα in human liver, in particular with respect to target gene regulation. To overcome this limitation, we collected precision cut liver slices (PCLS) from human subjects and studied the effect of PPARα activation on gene expression using whole genome expression profiling. In contrast to primary hepatocytes, PCLS mimic the multi-cellularity and structural organization of whole liver and thus represent a superior ex-vivo model system for human liver [27]. So far the use of PCLS for the study of nuclear receptors and specifically PPARα function has been limited [28–30]. Here we report the use of PCLS in combination with whole genome gene expression profiling to gain insight into PPARα-mediated gene regulation in human liver.

## Methods

### Collection of liver biopsies

In all patients a laparoscopic Roux-en-Y Gastric Bypass was performed under general anesthesia as treatment for their morbid obesity. Patients were instructed to fasten for solid foods and liquids starting at the night before surgery. During surgery a biopsy of the liver was obtained with the help of ultrasound dissection (UltraCision®). The biopsy was collected from the liver edge. The majority of the liver biopsies collected (*n* = 15) were immediately frozen in liquid nitrogen and stored at −80 °C. The liver biopsies targeted for slicing (*n* = 4) were immediately placed in ice-cold oxygenated Belzer UW Cold Storage Solution (Bridge to Life Ltd, Columbia, SC, USA) and quickly transferred to our laboratory for further processing of PCLS. Only macroscopically healthy livers were used for slicing. Biopsies were provided by the biobank of the Rijnstate hospital. Collection of biopsies for research purposes into the biobank was approved by the local institutional review board on behalf of the medical ethics review committee of the Radboud University Medical Centre. All patients signed informed consent for collection of the biopsy prior to surgery. Donor characteristics of the livers used for slicing are shown in Table 1.

**Table 1:** Donor characteristics of livers used for slicing

Patient	Gender	Age (years)	Body Mass Index (g/m2)
1	Female	34	41
2	Female	46	43
3	Female	39	35
4	Female	41	38

### Preparation and treatment of precision cut liver slices

PCLS were prepared and cultured as described previously [31]. 5 mm cylindrical liver cores were prepared with a surgical biopsy punch and sectioned to 200 μm slices using a Krumdieck tissue slicer (Alabama Research and Development, Munford, AL, USA) filled with carbonated KHB (pH 7.4, supplemented with 25 mM glucose). Liver slices were incubated in William’s E Medium (Gibco, Paisley, Scotland) supplemented with penicillin/streptomycin in 6-well plates at 37 °C/5 % CO_2_/80 % O_2_ under continuous shaking (70 rpm). Duplicate wells were used per donor with 3 liver slices per well. After 1 hour the media was replaced with fresh William’s E Medium in the presence or absence of Wy14643 (100 μM) dissolved in dimethyl sulfoxide (DMSO, final concentration 0.1 %). This concentration was chosen based on the affinity of human PPARα for Wy14643 [32]. After 24 h incubation, liver slices were snap-frozen in liquid nitrogen and stored in −80 °C for RNA isolation.

### Primary human hepatocytes

The treatment of primary human hepatocytes with Wy14643 has been previously described [26]. Briefly, human hepatocytes from six different donors were purchased from Lonza (Verviers, Belgium). Hepatocytes were isolated with two-step collagenase perfusion method. Cell viability was over 80 %. The cells were incubated for 24 h in the presence or absence of Wy14643 (50 μM) dissolved in DMSO, followed by RNA isolation.

### RNA isolation and qPCR

Total RNA was isolated using TRIzol reagent (Invitrogen). RNA integrity number was found to be above 7.1 for all samples suggesting that the human liver slices were of good quality. The RNA integrity number is based on a digital electropherogram generated using a Agilent bioanalyzer. It describes the degree of degradation of RNA with level 10 representing completely intact RNA. RNA was reverse transcribed using a iScript cDNA Synthesis Kit (Bio-Rad Laboratories BV, Veenendaal, The Netherlands). Real-time PCR was carried out using SensiMiX (Bioline) on a CFX 384 Bio-Rad thermal cycler (Bio-Rad). 36B4 was used as housekeeping gene. Primer sequences used are shown in Table 2.

**Table 2:** Primer sequences used for qPCR

	Primer Sequence	
Name	Forward	Reverse
36B4	CGGGAAGGCTGTGGTGCTG	GTGAACACAAAGCCCACATTCC
ANGPTL4	CACAGCCTGCAGACACAACTC	GGAGGCCAAACTGGCTTTGC
PLIN2	ATGGCATCCGTTGCAGTTGAT	GATGGTCTTCACACCGTTCTC
PDK4	TGGAGCATTTCTCGCGCTAC	ACAGGCAATTCTTGTCGCAAA
CPT1A	TCCAGTTGGCTTATCGTGGTG	CTAACGAGGGGTCGATCTTGG
VLDLR	GGTGAAAATGATTGTGACAGTGG	GTGAACTCGTCGGGACTACA

### Microarray analysis

Total RNA was purified with RNeasy Minikit columns (Qiagen) and RNA quality was assessed using RNA 6000 Nano chips on the Agilent 2100 Bioanalyzer (Agilent Technologies, Amsterdam, The Netherlands). Purified RNA (100 ng) was labeled with the Ambion WT expression kit (Invitrogen) and hybridized to an Affymetrix Human Gene 1.1 ST array plate (Affymetrix, Santa Clara, CA). Hybridization, washing, and scanning were carried out on an Affymetrix GeneTitan platform according to the instruction by the manufacturer. Arrays were normalized using the Robust Multiarray Average method [33, 34]. Probe sets were defined according to Dai et al. [35]. In this method probes are assigned to Entrez IDs as an unique gene identifier. The *P*-value for the effect of Wy14643 treatment were calculated using an Intensity-Based Moderated T-statistic (IBMT) [36]. The *q*-value was calculated as measure of significance for false discovery rate [37]. The microarray data for the human liver slices were submitted to Gene Expression Omnibus (GSE71731). The microarray data for the human primary hepatocytes have been previously submitted to Gene Expression Omnibus (GSE17251).

Gene set enrichment analysis (GSEA) was used to find enriched gene sets in the induced or suppressed genes [38]. Genes were ranked based on the paired IBMT-statistic and subsequently analyzed for over- or underrepresentation in predefined gene sets derived from Gene Ontology, KEGG, National Cancer Institute, PFAM, Biocarta, Reactome and WikiPathways pathway databases. Only gene sets consisting of more than 15 and fewer than 500 genes were taken into account. Statistical significance of GSEA results was determined using 1000 permutations.

## Results

**Fig. 1:**
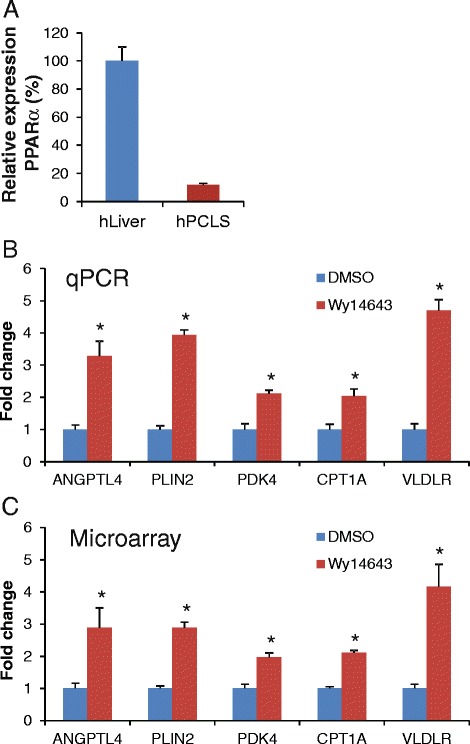
Classical PPARα targets genes are robustly induced by PPARα activation in human PCLS. **a** Expression level of PPARα in human liver biopsies (*n* = 15) and human PCLS (*n* = 5). **b** Gene expression changes of selected PPARα target genes in human PCLS in response to 24 h Wy14643 treatment as determined by quantitative real-time PCR. **c** Gene expression changes of the same genes according to microarray. Error bars represent SEM. Asterisk indicates statistically significant (*P* < 0.05, Student's *t*-test)

First, we determined the relative expression level of PPARα in PCLS in comparison with human liver. After correcting for the housekeeping gene 36B4, mRNA levels of PPARα in human PCLS after 24 h incubation were about ten-fold lower as compared to snap-frozen human liver biopsies (Fig. 1a). To verify that the human liver slices maintain their ability to respond to PPARα activation, we exposed the PCLS to 100 μM Wy14643, isolated total RNA and performed qPCR to determine the expression of a number of well-established PPARα target genes, including PLIN2, VLDLR, ANGPTL4, CPT1A and PDK4 (Fig. 1b). All PPARα target genes analyzed showed significant induction following Wy14643 treatment, indicating the validity of our model to study PPARα-mediated gene regulation.

**Fig. 2:**
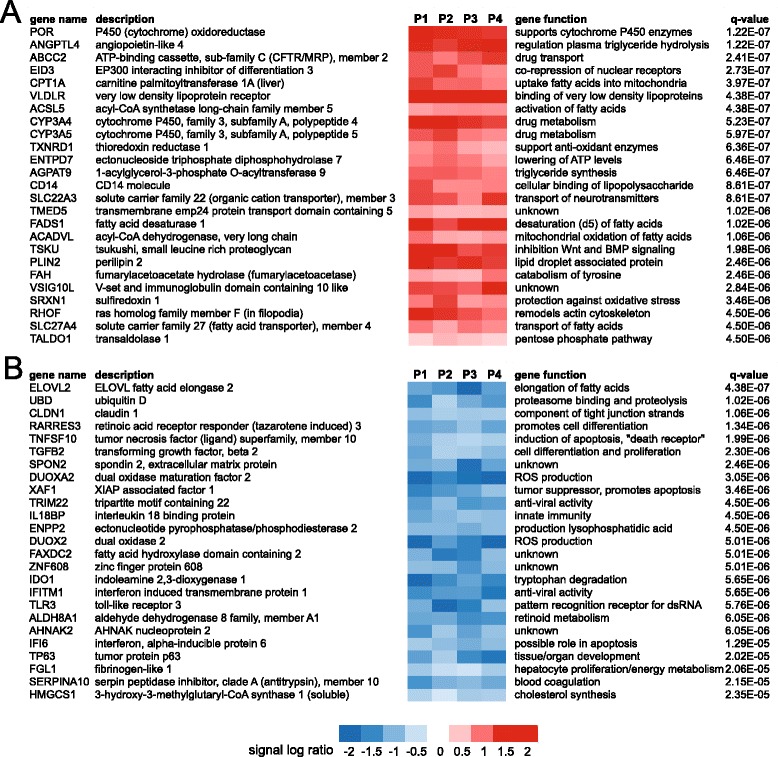
Top 25 genes induced or repressed by Wy14643 in human PCLS. Heatmap showing gene expression changes of the top 25 most significantly induced (**a**) and repressed (**b**) genes in human PCLS treated with Wy14643 for 24 h, as determined by microarray analysis. Genes were ranked based on statistical significance in the form of *q*-value (IBMT regularised paired *t*-test). P1 to P4 represent the four human subjects that donated a liver specimen for preparation of PCLS

To study the effect of PPARα activation on whole genome gene expression, we performed microarray analysis. Wy14643-induced changes in expression of the selected PPARα target genes were very similar between the microarray and qPCR analysis (Fig. 1c). Using a FDR *q*-value of 0.05 as cut-off (IBMT regularised paired *t*-test), the expression of 1282 genes (out of 19,654 genes on the array) was found to be significantly altered by Wy14643 treatment, of which 617 genes were upregulated and 665 genes were downregulated. The top 25 of most significantly induced genes, ranked according to statistical significance, are shown in Fig. 2a. The full list is available as Additional file 1. The list includes many well-known PPARα target genes involved in lipid metabolism (e.g. VLDLR, ACADVL, PLIN2, ANGPTL4, CPT1A), as well as many other genes covering a wide variety of biological functions. In addition, the list includes a number of genes with unknown function. Figure 2b shows the top 25 of most significantly repressed genes, many of which are related to immune function and inflammation. The full list of significantly downregulated genes is available as Additional file 2.

**Fig. 3:**
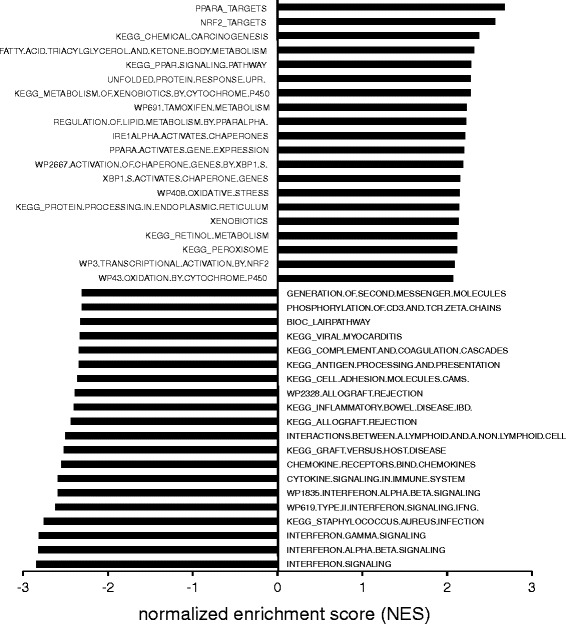
Top 20 gene sets induced or repressed by Wy14643 in human PCLS. The top 20 most significantly induced or repressed pathways in human PCLS in response to 24 h Wy14643 treatment were determined by gene set enrichment analysis. Gene sets were ranked according to normalized enrichment score (NES)

To gain better insight into the biological function of genes regulated by PPARα activation in human liver slices, we performed gene set enrichment analysis (GSEA). Pathways related to lipid metabolism or directly involving PPARα featured prominently among the gene sets induced by Wy14643 (Fig. 3). Also, several gene sets induced by Wy14643 were related to the unfolded protein response and UPR signaling by IRE1α and XBP1. Finally, we observed significant enrichment of genes related to oxidative stress and xenobiotic/drug metabolism. Gene sets downregulated by PPARα activation were all related to immune function and inflammation, illustrating a potent anti-inflammatory/immuno-suppressive action of PPARα in human liver.

An important goal of the present work was to test the suitability of liver slices as a model to study PPARα dependent gene regulation and compare it with other available model systems for human liver. To that end, we compared the whole genome expression profiles of human liver slices treated with Wy14643 with the expression profiles of primary human hepatocytes treated with Wy14643. The expression profiles of primary human hepatocytes have been previously published but were re-analyzed [26]. The primary human hepatocytes were treated for the same duration and microarray analysis was performed in the same laboratory on the same microarray platform.

**Fig. 4:**
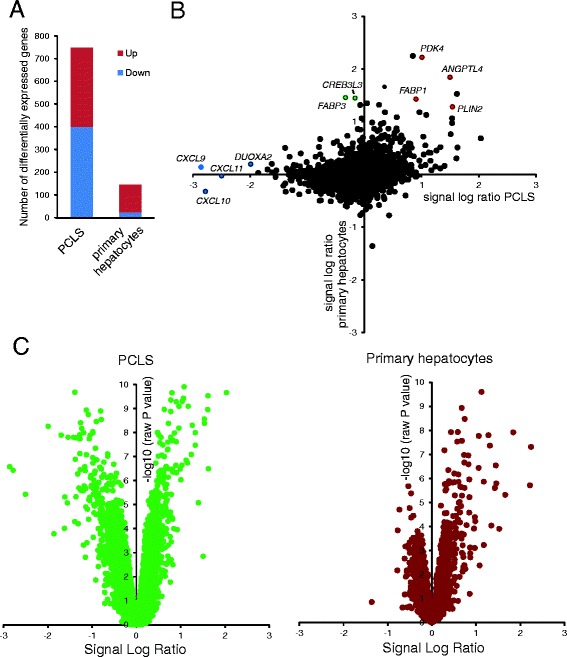
Comparative analysis of effect Wy14643 on gene expression in human PCLS and primary hepatocytes. **a** The number of differentially expressed genes in human PCLS and human primary hepatocytes in response to 24 h Wy14643 treatment in a common geneset of 17,351 genes was calculated based on a statistical significance cut-off of *P* < 0.001 (IBMT regularised paired *t*-test) and fold-change >1.20. Genes were separated according to up- or down-regulation. **b** Correlation plot showing changes in gene expression in response to Wy14643 (expressed as signal log ratio) in human PCLS (x-axis) and primary human hepatocytes (y-axis). Selected PPARα target genes commonly induced by Wy14643 in PCLS and hepatocytes are highlighted in red. Selected inflammation-related genes specifically repressed by Wy14643 in PCLS are highlighted in blue. Selected lipid metabolism-related genes specifically induced by Wy14643 in primary hepatocytes are highlighted in green. **c** Volcano plot showing relative changes in gene expression in response to Wy14643 (expressed as signal log ratio, x-axis) plotted against statistical significance (expressed as IBMT regularised paired *t*-test *P*-value, y-axis) for the PCLS and primary hepatocytes

The comparative analysis between primary human hepatocytes and human liver slices was performed on a common gene set of 17,351 genes. Using a statistical cut-off of *P* < 0.001 (IBMT regularised paired *t*-test) combined with a fold-change cut-off of 1.2, we found 347 genes to be upregulated in human liver slices, as compared to 121 genes in the hepatocytes (Fig. 4a), with an overlap of 47 genes. Remarkably, whereas 401 genes were downregulated by Wy14643 in the liver slices, only 25 genes were downregulated by Wy14643 in the human hepatocytes, with an overlap of only 4 genes. The data indicate that the human liver slices are much more sensitive towards especially downregulation of gene expression by Wy14643 as compared to human hepatocytes. This notion was further supported by correlation analysis in the form of a scatter plot (Fig. 4b). The upper right portion of the scatter plot was well filled (Fig. 4b), reflecting common induction by Wy14643 in PCLS and primary hepatocytes, including well-known PPARα targets such as FABP1, PLIN2, PDK4 and ANGPTL4. In contrast, the lower left portion of the scatter plot was much less filled (Fig. 4b), reflecting little agreement between PCLS and primary hepatocytes with respect to downregulation of gene expression by Wy14643. In fact, many genes were markedly downregulated by Wy14643 in the liver slices but showed no change in expression in the hepatocytes. Nearly all genes conforming to this type of expression were involved in immune-related function, as illustrated by the chemokines CXCL9, CXCL10, and CXCL11. Interestingly, a number of genes was explicitly induced by PPARα activation in primary hepatocytes but not in liver slices, including FABP3 as well as CREB3L3, a possible mediator of the stimulatory effects of PPARα on hepatic gene expression [11]. Subsequent analysis by Volcano plot confirmed the overall more pronounced effect of PPARα activation on gene expression in liver slices as compared to primary hepatocytes and also corroborated the relatively minor down-regulation of gene expression by PPARα activation in primary hepatocytes (Fig. 4c).

**Fig. 5:**
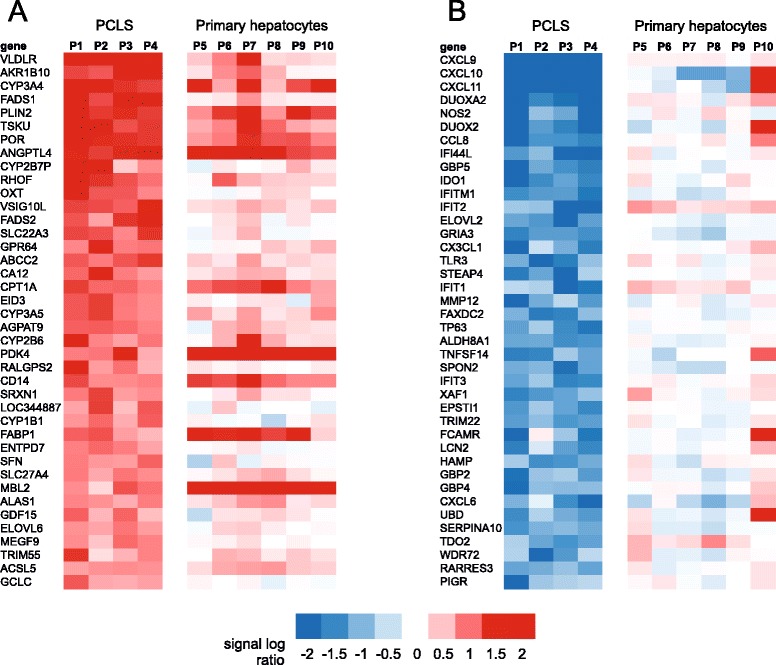
Comparative heatmap analysis of effect Wy14643 on gene expression in human PCLS and primary hepatocytes. Genes that were statistically significantly regulated by Wy14643 in human PCLS (*P* < 0.001, IBMT regularised paired *t*-test) were ranked according to fold-change in expression. The top 40 genes with highest fold-induction (**a**) or fold-repression (**b**) are shown. Expression changes of the same gene set in primary hepatocytes is shown in the right panel. P1 to P4 represent the four human subjects that donated a liver specimen for preparation of PCLS. P5 to P10 represent the six human subjects that donated a liver specimen for preparation of human hepatocytes

To further investigate this observation, we took the 40 genes most highly induced or repressed by Wy14643 in the liver slices (*P* < 0.001 and ranked according to fold-change) and studied the response to Wy14643 of the same genes in the primary hepatocytes. Whereas the majority of genes induced by Wy14643 in the liver slices were also induced by Wy14643 in the hepatocytes (Fig. 5a)—as illustrated by the common induction of well-known targets such as FABP1, PLIN2, FADS1 and ANGPTL4—few genes that were downregulated by Wy14643 in liver slices were also consistently downregulated by Wy14643 in primary hepatocytes (Fig. 5b). Also, the magnitude of gene suppression by Wy14643 was generally much less pronounced in primary hepatocytes. Confirming the GSEA results, the majority of the most highly repressed genes were related to immunity and inflammation, including the aforementioned chemokines (CXCL9-11, CCL8, CX3CL1, CXCL6), interferon γ-induced genes (IFITM1, IFIT1, IFIT2, IFIT3) and other immune-related genes (TLR3, NOS2, and LCN2).

**Fig. 6:**
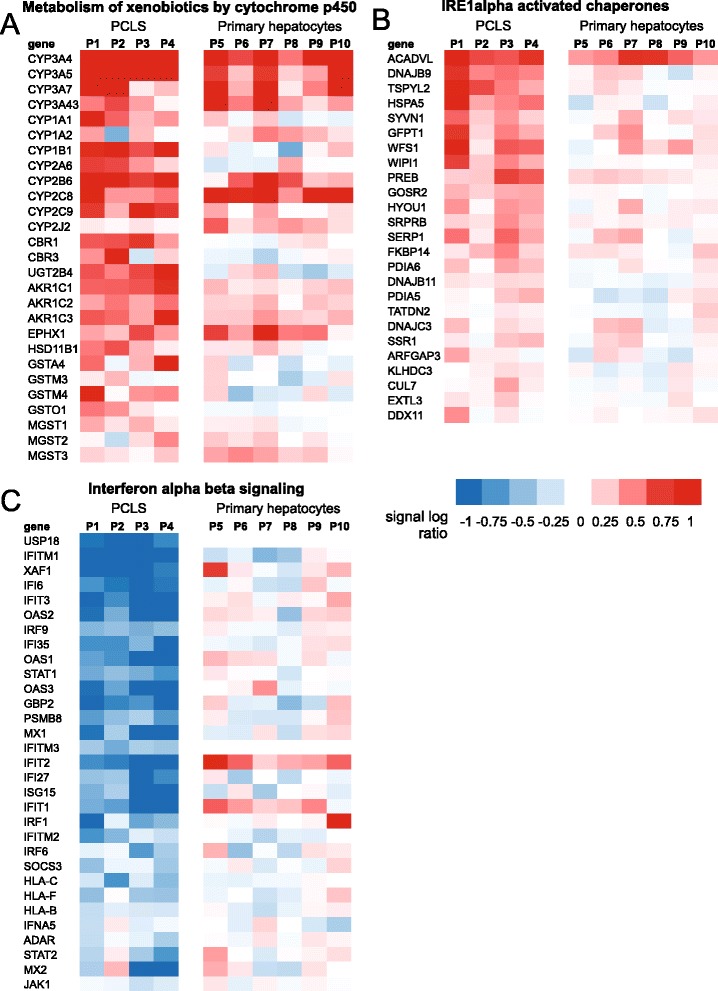
Regulation of selected gene sets by Wy14643 in human PCLS and primary hepatocytes. Heatmap showing gene expression changes of enriched genes that are part of the gene sets “metabolism of xenobiotics by cytochrome P450” (**a**), “IRE1a activated chaperones” (**b**), and “Interferon alpha beta signaling” (**c**) in human PCLS and primary hepatocytes. P1 to P4 represent the four human subjects that donated a liver specimen for preparation of PCLS. P5 to P10 represent the six human subjects that donated a liver specimen for preparation of human hepatocytes

To further explore the similarity in gene regulation by Wy14643 between PCLS and primary hepatocytes, we took the enriched genes within the positively or negatively enriched gene sets “IRE1α activated chaperones”, “metabolism of xenobiotics by cytochrome P450”, and “Interferon alpha beta signaling” (Fig. 3), and compared gene expression changes between PCLS and primary hepatocytes. Induction of most genes that are part of “metabolism of xenobiotics by cytochrome P450” was more pronounced in PCLS than in primary hepatocytes but reasonably well conserved between the two model systems (Fig. 6a). A limited number of genes (i.e. CYP2J2) showed higher fold-inductions in the primary hepatocytes as compared to PCLS. Induction of genes part of “IRE1α activated chaperones” was generally less pronounced in comparison with genes part of “metabolism of xenobiotics by cytochrome P450”, and was relatively weakly conserved between PCLS and primary hepatocytes (Fig. 6b). An exception is ACADVL. However, the inclusion of ACADVL (very long chain acyl-CoA dehydrogenase = fatty acid oxidation) within “IRE1α activated chaperones” may be questioned. Consistent with the other data showing potent downregulation of immune- and inflammation-related genes by PPARα activation in PCLS but not primary hepatocytes, suppression of genes part of “Interferon alpha beta signaling” was exclusively observed in PCLS (Fig. 6c).

**Fig. 7:**
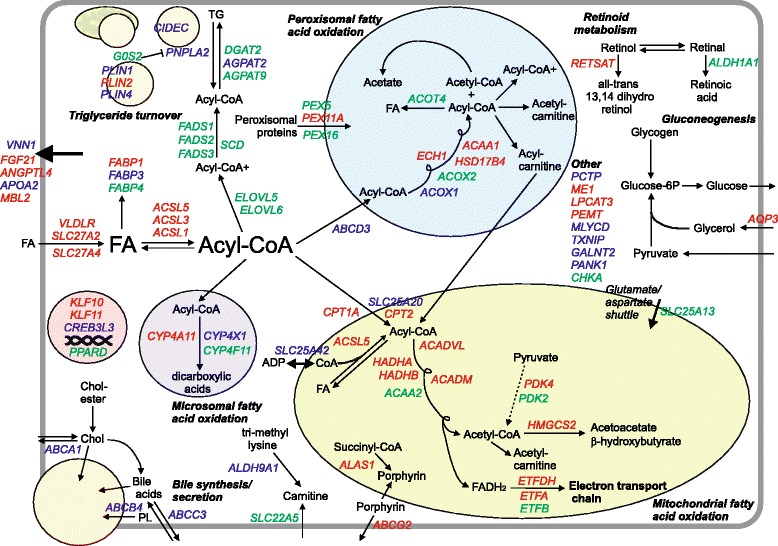
Overview of regulation of lipid metabolism by PPARα in human liver. A detailed overview map was created of metabolic genes upregulated by PPARα in human liver based on transcriptomics analysis of human PCLS and primary hepatocytes treated with Wy14643. Genes indicated in red are significantly induced by Wy14643 in human PCLS and primary hepatocytes. Genes indicated in green are significantly induced by Wy14643 in human PCLS but not primary hepatocytes. Genes indicated in blue are significantly induced by Wy14643 in human primary hepatocytes but not PCLS. Statistical significance was determined by IBMT regularised paired *t*-test (*P* < 0.01)

Finally, we used the microarray data of the human primary hepatocytes and human PCLS treated with Wy14643 to generate a detailed gene map of known and putative PPARα target genes involved in lipid metabolic pathways (Fig. 7). The map illustrates that regulation of bile acid synthesis and secretion, which is a well-established PPARα target pathway in mouse, was only evident in primary hepatocytes and not in liver slices. Conversely, genes involved in fatty acid elongation and desaturation were clearly induced by PPARα activation in human liver slices but not in primary hepatocytes.

## Discussion

The main conclusion of our study is that induction of gene expression by PPARα activation is generally well captured and shows significant overlap between human liver slices and primary human hepatocytes, showing consistent upregulation of genes involved in lipid and xenobiotic metabolism in the two model systems. In contrast, downregulation of gene expression by PPARα activation is almost exclusively observed in human liver slices. A previous study comparing mouse primary hepatocytes, mouse liver slices and mouse liver reached a similar conclusion [30]. Overall, our data indicate that human PCLS are a superior model to study PPARα-dependent gene regulation and PPARα functions in human liver.

As indicated above, PPARα activation caused major downregulation of gene expression in human liver slices but not in primary hepatocytes. A key difference between primary hepatocytes and liver slices is that the primary hepatocyte culture consists of only hepatocytes, whereas the liver slices contain other cell types, including stellate cells and Kupffer cells. A large portion of the downregulated genes and pathways in the liver slices was found to be connected to the immune system. Genes that were highly repressed upon PPARα activation included several chemokines (e.g. CXCL9-11, CCL8, CX3CL1, CXCL6), interferon γ-induced genes (e.g. IFITM1, IFIT1, IFIT2, IFIT3) and numerous other immune-related genes (e.g. TLR3, NOS2, and LCN2). Downregulation of gene expression is unlikely to be mediated by PPARα present in non-parenchymal cells, as PPARα expression in these cells is very low [30, 39]. Instead we prefer a scenario in which the immuno-suppressive action of PPARα activation in hepatocytes is dependent on (inflammatory) signals emanating from non-parenchymal cells. Indeed, downregulation of inflammatory gene expression in primary hepatocytes and mouse liver by PPARα activation is sensitive to the presence of pro-inflammatory stimuli [40, 41]. Previously, we and others already demonstrated that Kupffer cells promote fat storage in hepatocytes by releasing inflammatory signals such as IL-1β, causing downregulation of PPARα gene expression [42, 43]. Overall, these data suggest that the full scope of functions of PPARα in hepatocytes is critically dependent on the interaction with other liver cell types. It is of interest to note that recently the anti-inflammatory action of PPARα in mouse liver was unequivocally attributed to the ability of PPARα to interact with other transcription factor pathways—a property referred to as transrepression—independent of the DNA-binding ability of PPARα, as shown in the context of steatohepatitis [44].

Despite the clear clinical efficacy of fibrates towards lowering of circulating triglycerides, the lack of peroxisome proliferation in human primary hepatocytes following PPARα activation has fed the idea that humans are largely insensitive to peroxisome-proliferator-induced hepatic effects and that the functional role of PPARα in human liver may be limited [45–47]. Subsequent whole genome expression profiling studies in primary human hepatocytes have mostly discounted these notions [25, 26]. We found that PPARα is highly expressed in human liver with Ct values ranging from 22 to 26, which is similar to the values observed in mouse liver (data not shown). Importantly, despite the markedly lower expression of PPARα in human PCLS as compared to human liver, activation of PPARα in PCLS caused pronounced upregulation or downregulation of numerous genes, including many known PPARα targets, strongly supporting the functionality of PPARα in human PCLS and giving strong credibility to an important in vivo role of PPARα in human liver. Recently, it was found that human liver PPARα gene expression correlates negatively with the severity of steatohepatitis and with measures of insulin resistance. Furthermore, histological improvement in a follow-up biopsy was associated with increased expression of PPARα and its target genes, suggesting that PPARα is involved in and may be therapeutically targeted for human steatohepatitis [48].

The majority of gene sets enriched among the upregulated genes were related to lipid and xenobiotic metabolism, which are well-established target pathways of PPARα. Intriguingly, several highly enriched gene sets were part of the unfolded protein response and IRE1α-XBP1 signaling, two key factors involved in governing UPR. Currently, there are no published data linking PPARα to IRE1α-XBP1 signaling and regulation of UPR, though it has been observed that PPARα is involved in regulating proteome maintenance by inducing numerous heat shock proteins [49]. Surprisingly, recently it was demonstrated that IRE1α-XBP1 signaling leads to activation of PPARα via direct binding of XBP1s to the promoter of PPARα, thereby stimulating mitochondrial β-oxidation and ketogenesis [50]. Thus, there appear to be reciprocal interactions between PPARα and UPR. The wider biological framework for regulation of UPR requires further clarification.

Our analysis reveals differential regulation of a number of PPARα targets between liver slices and primary hepatocytes. For instance, VNN1 was significantly upregulated by PPARα activation in primary hepatocytes but showed no change in expression in liver slices. Conversely, expression of FADS2 was significantly increased in human liver slices but showed no change in primary hepatocytes. The differential regulation of specific PPARα target genes by Wy14643 between primary hepatocytes and liver slices may be a reflection of the different cellular context in the two models systems, with non-hepatocytes potentially exerting a stimulatory or inhibitory influence on PPARα-dependent gene induction. However, it should be realized that for a number of genes the seemingly differential regulation may reflect a quantitative difference rather than a true qualitative difference. For example, SLC25A20 was induced significantly by 1.58-fold in primary hepatocytes as compared to a non-significant 1.34-fold induction in liver slices, barely missing the statistical significance cut-off.

Our analysis yielded a number of relatively poorly characterized genes that showed a pronounced and consistent upregulation upon PPARα activation in the two human liver model systems. These include TSKU, RHOF, CA12 and VSIG10L. Interestingly, many genes that were found to be induced by PPARα in early microarray analyses and which did not have an assigned function at the time were later shown to be involved in some aspect of lipid metabolism. Accordingly, it can be hypothesized that the above mentioned genes as well as other poorly characterized genes that are commonly induced by Wy14643 in various liver model systems may be directly or indirectly connected to lipid metabolism.

The comparative microarray analysis of the effect of PPARα activation in primary human hepatocytes and human liver slices is somewhat hampered by a number of different factors, including the use of different types of Affymetrix gene chips, different human donors, and an unequal number of biological replicates per group. However, treatments of liver slices and hepatocytes were carried out for the same duration and with the same PPARα agonist. Furthermore, RNA was isolated and labeled via the same technique, hybridizations were performed on the same platform by the same technician, and the microarray data were processed in parallel using the same analysis methods.

On a final note, the data collected in this paper were added to a publicly available overview map of known (lipid) metabolic genes upregulated by PPARα in human liver (accessible via: http://en.wikipedia.org/wiki/Peroxisome_proliferator-activated_receptor_alpha), which was generated largely by using published transcriptome datasets.

## Conclusion

In conclusion, our paper demonstrates the suitability and superiority of PCLS over primary human hepatocytes for studying the functional role of PPARα in human liver. Our data underscore the major role of PPARα in regulation of hepatic lipid and xenobiotic metabolism and reveal a marked immuno-suppressive/anti-inflammatory effect of PPARα in human liver that may be therapeutically relevant for NAFLD. The data add to our growing understanding of the critical role of PPARα in gene regulation in human liver.

## Additional files

Additional file 1:
**Full list of significantly induced genes by Wy14643 in human PCLS.** (PDF 211 kb) Additional file 2:
**Full list of significantly repressed genes by Wy14643 in human PCLS.** (PDF 228 kb)

## Abbreviations

PPARα: Peroxisome proliferator activated receptor alpha
PCLS: Precision cut liver slices
NAFLD: Non-alcoholic fatty liver disease
DMSO: Dimethyl sulfoxide
GSEA: Gene set enrichment analysis
IBMT: Intensity-Based Moderated T-statistic
