# Supplementary material for: The impact of PPARα activation on whole genome gene expression in human precision cut liver slices
Source: BMC Genomics. 2015 Oct 8;16:760. doi: 10.1186/s12864-015-1969-3 (PMC4599789; doi:10.1186/s12864-015-1969-3)
Supplement: Additional file 1: — Full list of significantly induced genes by Wy14643 in human PCLS. (PDF 211 kb) [file 12864_2015_1969_MOESM1_ESM.pdf]

Entrez ID	gene name	fold change	q-value	description
7436	VLDLR	4.11	4.38E-07	very low density lipoprotein receptor
57016	AKR1B10	3.09	6.09E-05	aldo-keto reductase family 1, member B10 (aldose reductase)
1576	CYP3A4	3.07	5.23E-07	cytochrome P450, family 3, subfamily A, polypeptide 4
3992	FADS1	3.06	1.02E-06	fatty acid desaturase 1
123	PLIN2	2.91	2.46E-06	perilipin 2
25987	TSKU	2.90	1.98E-06	tsukushi, small leucine rich proteoglycan
5447	POR	2.90	1.22E-07	P450 (cytochrome) oxidoreductase
1551	CYP3A7	2.85	2.50E-02	cytochrome P450, family 3, subfamily A, polypeptide 7
51129	ANGPTL4	2.82	1.22E-07	angiopoietin-like 4
1556	CYP2B7P	2.64	6.79E-04	cytochrome P450, family 2, subfamily B, polypeptide 7, pseudogene
54509	RHOF	2.52	4.50E-06	ras homolog family member F (in filopodia)
5020	OXT	2.52	9.73E-06	oxytocin/neurophysin I prepropeptide
147645	VSIG10L	2.40	2.84E-06	V-set and immunoglobulin domain containing 10 like
9415	FADS2	2.37	2.15E-05	fatty acid desaturase 2
6581	SLC22A3	2.26	8.61E-07	solute carrier family 22 (organic cation transporter), member 3
10149	GPR64	2.23	4.02E-05	G protein-coupled receptor 64
1244	ABCC2	2.23	2.41E-07	ATP-binding cassette, sub-family C (CFTR/MRP), member 2
771	CA12	2.18	3.21E-05	carbonic anhydrase XII
1374	CPT1A	2.12	3.97E-07	carnitine palmitoyltransferase 1A (liver)
493861	EID3	2.10	2.73E-07	EP300 interacting inhibitor of differentiation 3
1577	CYP3A5	2.09	5.97E-07	cytochrome P450, family 3, subfamily A, polypeptide 5
84803	AGPAT9	2.05	6.46E-07	1-acylglycerol-3-phosphate O-acyltransferase 9
1555	CYP2B6	2.04	4.78E-05	cytochrome P450, family 2, subfamily B, polypeptide 6
5166	PDK4	2.01	1.28E-04	pyruvate dehydrogenase kinase, isozyme 4
55103	RALGPS2	1.99	6.09E-05	Ral GEF with PH domain and SH3 binding motif 2
929	CD14	1.95	8.61E-07	CD14 molecule
140809	SRXN1	1.95	3.46E-06	sulfiredoxin 1
344887	LOC344887	1.90	4.01E-03	NmrA-like family domain containing 1 pseudogene
1545	CYP1B1	1.87	7.74E-05	cytochrome P450, family 1, subfamily B, polypeptide 1
2168	FABP1	1.87	7.58E-06	fatty acid binding protein 1, liver
57089	ENTPD7	1.87	6.46E-07	ectonucleoside triphosphate diphosphohydrolase 7
2810	SFN	1.86	4.82E-05	stratifin
10999	SLC27A4	1.82	4.50E-06	solute carrier family 27 (fatty acid transporter), member 4
4153	MBL2	1.80	3.76E-03	mannose-binding lectin (protein C) 2, soluble
211	ALAS1	1.78	6.88E-06	aminolevulinate, delta-, synthase 1
135656	DPCR1	1.77	4.24E-02	diffuse panbronchiolitis critical region 1
9518	GDF15	1.77	7.30E-05	growth differentiation factor 15
79071	ELOVL6	1.77	1.94E-03	ELOVL fatty acid elongase 6
1955	MEGF9	1.76	6.13E-05	multiple EGF-like-domains 9
84675	TRIM55	1.75	1.78E-03	tripartite motif containing 55
51703	ACSL5	1.75	4.38E-07	acyl-CoA synthetase long-chain family member 5
2729	GCLC	1.75	7.08E-06	glutamate-cysteine ligase, catalytic subunit
28449	IGHV3-13	1.75	2.94E-02	immunoglobulin heavy variable 3-13
54210	TREM1	1.73	3.69E-04	triggering receptor expressed on myeloid cells 1
2151	F2RL2	1.73	3.11E-02	coagulation factor II (thrombin) receptor-like 2
10786	SLC17A3	1.73	9.39E-03	solute carrier family 17 (organic anion transporter), member 3
6974	TRGV2	1.72	4.52E-02	T cell receptor gamma variable 2
7296	TXNRD1	1.72	6.36E-07	thioredoxin reductase 1
4835	NQO2	1.72	5.65E-06	NAD(P)H dehydrogenase, quinone 2
37	ACADVL	1.72	1.06E-06	acyl-CoA dehydrogenase, very long chain
23657	SLC7A11	1.71	1.25E-03	solute carrier family 7 (anionic amino acid transporter light chain, xc- system), member 11
552	AVPR1A	1.71	1.56E-03	arginine vasopressin receptor 1A
3131	HLF	1.70	6.58E-03	hepatic leukemia factor
11069	RAPGEF4	1.70	1.78E-03	Rap guanine nucleotide exchange factor (GEF) 4
10058	ABCB6	1.70	6.09E-05	ATP-binding cassette, sub-family B (MDR/TAP), member 6
1591	CYP24A1	1.66	3.37E-02	cytochrome P450, family 24, subfamily A, polypeptide 1
2184	FAH	1.66	2.46E-06	fumarylacetoacetate hydrolase (fumarylacetoacetase)
13	AADAC	1.65	1.04E-04	arylacetamide deacetylase
8431	NR0B2	1.65	1.18E-02	nuclear receptor subfamily 0, group B, member 2
3148	HMGB2	1.65	1.24E-03	high mobility group box 2
89866	SEC16B	1.64	2.22E-03	SEC16 homolog B (S. cerevisiae)
7363	UGT2B4	1.64	7.62E-06	UDP glucuronosyltransferase 2 family, polypeptide B4
23189	KANK1	1.64	1.40E-05	KN motif and ankyrin repeat domains 1
990	CDC6	1.64	2.47E-02	cell division cycle 6
2730	GCLM	1.63	1.44E-05	glutamate-cysteine ligase, modifier subunit
54346	UNC93A	1.63	1.38E-03	unc-93 homolog A (C. elegans)
57761	TRIB3	1.62	5.48E-04	tribbles pseudokinase 3
7378	UPP1	1.62	1.47E-03	uridine phosphorylase 1
168620	BHLHA15	1.61	1.30E-03	basic helix-loop-helix family, member a15
1528	CYB5A	1.61	3.69E-04	cytochrome b5 type A (microsomal)
2167	FABP4	1.61	4.10E-02	fatty acid binding protein 4, adipocyte
1559	CYP2C9	1.61	1.76E-04	cytochrome P450, family 2, subfamily C, polypeptide 9
4862	NPAS2	1.60	5.28E-03	neuronal PAS domain protein 2
6584	SLC22A5	1.60	3.82E-03	solute carrier family 22 (organic cation/carnitine transporter), member 5
2181	ACSL3	1.60	5.26E-05	acyl-CoA synthetase long-chain family member 3
54518	APBB1IP	1.60	6.47E-04	amyloid beta (A4) precursor protein-binding, family B, member 1 interacting protein
1891	ECH1	1.59	5.33E-05	enoyl CoA hydratase 1, peroxisomal
65084	TMEM135	1.59	9.41E-04	transmembrane protein 135
1645	AKR1C1	1.58	6.09E-05	aldo-keto reductase family 1, member C1
11153	FICD	1.58	2.38E-04	FIC domain containing
11200	CHEK2	1.58	4.83E-02	checkpoint kinase 2
54551	MAGEL2	1.58	3.94E-02	MAGE-like 2
2852	GPER1	1.57	4.80E-03	G protein-coupled estrogen receptor 1
79729	SH3D21	1.57	7.73E-04	SH3 domain containing 21
164832	LONRF2	1.57	1.39E-02	LON peptidase N-terminal domain and ring finger 2
8644	AKR1C3	1.57	3.76E-03	aldo-keto reductase family 1, member C3
8462	KLF11	1.57	1.86E-04	Kruppel-like factor 11
4882	NPR2	1.56	3.72E-03	natriuretic peptide receptor 2
84263	HSDL2	1.55	2.41E-05	hydroxysteroid dehydrogenase like 2
50999	TMED5	1.55	1.02E-06	transmembrane emp24 protein transport domain containing 5
873	CBR1	1.54	1.04E-04	carbonyl reductase 1
6319	SCD	1.54	1.17E-03	stearoyl-CoA desaturase (delta-9-desaturase)
55532	SLC30A10	1.54	8.22E-05	solute carrier family 30, member 10
1271	CNTFR	1.53	5.22E-03	ciliary neurotrophic factor receptor
51076	CUTC	1.53	4.39E-04	cutC copper transporter
133746	JMY	1.53	9.05E-03	junction mediating and regulatory protein, p53 cofactor
3024	HIST1H1A	1.53	5.74E-03	histone cluster 1, H1a
193629	LINC00189	1.52	9.60E-03	long intergenic non-protein coding RNA 189
84883	AIFM2	1.52	1.30E-04	apoptosis-inducing factor, mitochondrion-associated, 2
64061	TSPYL2	1.52	1.55E-03	TSPY-like 2

5226	PGD	1.51	9.62E-06	phosphogluconate dehydrogenase
7466	WFS1	1.51	1.59E-03	Wolfram syndrome 1 (wolframin)
3613	IMPA2	1.51	1.47E-02	inositol(myo)-1(or 4)-monophosphatase 2
91614	DEPDC7	1.50	3.03E-03	DEP domain containing 7
275	AMT	1.50	1.20E-03	aminomethyltransferase
5652	PRSS8	1.50	9.97E-03	protease, serine, 8
1647	GADD45A	1.50	1.00E-02	growth arrest and DNA-damage-inducible, alpha
4189	DNAJB9	1.50	4.13E-04	DnaJ (Hsp40) homolog, subfamily B, member 9
79631	EFTUD1	1.49	1.39E-02	elongation factor Tu GTP binding domain containing 1
65124	SOWAHC	1.49	1.28E-03	sosondowah ankyrin repeat domain family member C
161436	EML5	1.49	1.55E-02	echinoderm microtubule associated protein like 5
3309	HSPA5	1.49	2.20E-05	heat shock 70kDa protein 5 (glucose-regulated protein, 78kDa)
51083	GAL	1.49	2.56E-02	galanin/GMAP prepropeptide
1376	CPT2	1.49	7.56E-03	carnitine palmitoyltransferase 2
10110	SGK2	1.48	3.63E-02	serum/glucocorticoid regulated kinase 2
26291	FGF21	1.48	3.54E-02	fibroblast growth factor 21
1558	CYP2C8	1.48	6.39E-05	cytochrome P450, family 2, subfamily C, polypeptide 8
2936	GSR	1.47	5.65E-06	glutathione reductase
10166	SLC25A15	1.47	5.23E-03	solute carrier family 25 (mitochondrial carrier /// ornithine transporter) member 15
83667	SESN2	1.47	4.17E-03	sestrin 2
83699	SH3BGRL2	1.47	3.83E-03	SH3 domain binding glutamate-rich protein like 2
734	OSGIN2	1.47	8.41E-04	oxidative stress induced growth inhibitor family member 2
132299	OCIAD2	1.47	1.48E-04	OCIA domain containing 2
114038	LINC00313	1.46	6.80E-03	long intergenic non-protein coding RNA 313
54825	CDHR2	1.45	1.27E-03	cadherin-related family member 2
444	ASPH	1.45	1.04E-04	aspartate beta-hydroxylase
3242	HPD	1.45	7.99E-06	4-hydroxyphenylpyruvate dioxygenase
51117	COQ4	1.45	6.58E-03	coenzyme Q4
144423	GLT1D1	1.45	1.31E-03	glycosyltransferase 1 domain containing 1
6505	SLC1A1	1.45	3.87E-03	solute carrier family 1 (neuronal/epithelial high affinity glutamate transporter, system Xag), member 1
84243	ZDHHC18	1.45	7.30E-05	zinc finger, DHHC-type containing 18
9540	TP53I3	1.44	9.80E-03	tumor protein p53 inducible protein 3
8517	IKBKG	1.44	3.37E-03	inhibitor of kappa light polypeptide gene enhancer in B-cells, kinase gamma
8800	PEX11A	1.44	8.67E-03	peroxisomal biogenesis factor 11 alpha
201161	CENPV	1.44	1.23E-02	centromere protein V
389432	SAMD5	1.44	2.20E-03	sterile alpha motif domain containing 5
54884	RETSAT	1.43	3.62E-03	retinol saturase (all-trans-retinol 13,14-reductase)
730102	LOC730102	1.43	2.76E-02	quinone oxidoreductase-like protein 2 pseudogene
1548	CYP2A6	1.43	3.35E-04	cytochrome P450, family 2, subfamily A, polypeptide 6
2632	GBE1	1.43	9.92E-05	glucan (1,4-alpha-), branching enzyme 1
127845	GOLT1A	1.43	7.42E-03	golgi transport 1A
22949	PTGR1	1.43	1.36E-04	prostaglandin reductase 1
9709	HERPUD1	1.42	9.37E-03	homocysteine-inducible, endoplasmic reticulum stress-inducible, ubiquitin-like domain member 1
5238	PGM3	1.42	7.30E-05	phosphoglucomutase 3
114883	OSBPL9	1.42	5.01E-06	oxysterol binding protein-like 9
7498	XDH	1.42	1.13E-02	xanthine dehydrogenase
8501	SLC43A1	1.42	1.98E-03	solute carrier family 43 (amino acid system L transporter), member 1
54978	SLC35F6	1.42	2.47E-03	solute carrier family 35, member F6
51302	CYP39A1	1.42	1.21E-02	cytochrome P450, family 39, subfamily A, polypeptide 1
133686	NADK2	1.42	4.50E-04	NAD kinase 2, mitochondrial
25998	IBTK	1.41	2.35E-05	inhibitor of Bruton agammaglobulinemia tyrosine kinase
4337	MOCS1	1.41	6.77E-03	molybdenum cofactor synthesis 1
3690	ITGB3	1.41	5.11E-04	integrin, beta 3 (platelet glycoprotein IIIa, antigen CD61)
55323	LARP6	1.41	2.16E-03	La ribonucleoprotein domain family, member 6
79605	PGBD5	1.41	2.11E-02	piggyBac transposable element derived 5
65009	NDRG4	1.41	3.79E-02	NDRG family member 4
152189	CMTM8	1.40	1.97E-03	CKLF-like MARVEL transmembrane domain containing 8
30061	SLC40A1	1.40	3.68E-02	solute carrier family 40 (iron-regulated transporter), member 1
79922	MRM1	1.40	4.56E-02	mitochondrial rRNA methyltransferase 1 homolog (S. cerevisiae)
51559	NT5DC3	1.40	2.51E-02	5'-nucleotidase domain containing 3
54893	MTMR10	1.40	6.83E-04	myotubularin related protein 10
343099	CCDC18	1.40	6.00E-03	coiled-coil domain containing 18
51533	PHF7	1.40	6.39E-03	PHD finger protein 7
10162	LPCAT3	1.40	1.45E-02	lysophosphatidylcholine acyltransferase 3
6888	TALDO1	1.39	4.50E-06	transaldolase 1
2110	ETFDH	1.39	8.52E-04	electron-transferring-flavoprotein dehydrogenase
997	CDC34	1.39	3.37E-03	cell division cycle 34
10486	CAP2	1.39	3.43E-03	CAP, adenylate cyclase-associated protein, 2 (yeast)
23327	NEDD4L	1.39	1.30E-03	neural precursor cell expressed, developmentally down-regulated 4-like, E3 ubiquitin protein ligase
847	CAT	1.39	1.91E-04	catalase
89894	TMEM116	1.39	1.75E-02	transmembrane protein 116
345757	FAM174A	1.39	1.00E-02	family with sequence similarity 174, member A
220108	FAM124A	1.39	3.84E-02	family with sequence similarity 124A
124512	METTL23	1.39	1.38E-02	methyltransferase like 23
4925	NUCB2	1.39	8.11E-03	nucleobindin 2
10113	PREB	1.38	1.25E-02	prolactin regulatory element binding
57406	ABHD6	1.38	1.32E-02	abhydrolase domain containing 6
51567	TDP2	1.38	2.79E-04	tyrosyl-DNA phosphodiesterase 2
84719	LINC00260	1.38	2.72E-02	long intergenic non-protein coding RNA 260
56605	ERO1LB	1.38	1.76E-03	ERO1-like beta (S. cerevisiae)
64093	SMOC1	1.38	1.04E-02	SPARC related modular calcium binding 1
63874	ABHD4	1.38	4.43E-04	abhydrolase domain containing 4
1646	AKR1C2	1.38	2.78E-04	aldo-keto reductase family 1, member C2
100272147	CMC4	1.38	1.15E-02	C-x(9)-C motif containing 4
2108	ETFA	1.38	1.38E-04	electron-transfer-flavoprotein, alpha polypeptide
3708	ITPR1	1.38	8.27E-04	inositol 1,4,5-trisphosphate receptor, type 1
11001	SLC27A2	1.38	1.29E-03	solute carrier family 27 (fatty acid transporter), member 2
57149	LYRM1	1.38	2.82E-03	LYR motif containing 1
2673	GFPT1	1.38	2.35E-03	glutamine--fructose-6-phosphate transaminase 1
112849	L3HYPDH	1.38	2.11E-02	L-3-hydroxyproline dehydratase (trans-)
9601	PDIA4	1.38	1.20E-03	protein disulfide isomerase family A, member 4
100033432	SNORD116-21	1.37	3.54E-02	small nucleolar RNA, C/D box 116-21
10449	ACAA2	1.37	7.03E-04	acetyl-CoA acyltransferase 2
8856	NR1I2	1.37	1.17E-02	nuclear receptor subfamily 1, group I, member 2
10891	PPARGC1A	1.37	3.61E-03	peroxisome proliferator-activated receptor gamma, coactivator 1 alpha
257364	SNX33	1.37	1.03E-02	sorting nexin 33
29948	OSGIN1	1.37	7.07E-04	oxidative stress induced growth inhibitor 1
101928229	NA	1.37	1.96E-02	NA
11080	DNAJB4	1.37	3.84E-02	DnaJ (Hsp40) homolog, subfamily B, member 4
55062	WIPI1	1.37	4.74E-04	WD repeat domain, phosphoinositide interacting 1
5603	MAPK13	1.37	3.20E-02	mitogen-activated protein kinase 13

9871	SEC24D	1.37	9.59E-04	SEC24 family member D
22885	ABLIM3	1.37	1.30E-03	actin binding LIM protein family, member 3
84561	SLC12A8	1.37	4.05E-03	solute carrier family 12, member 8
79746	ECHDC3	1.37	4.97E-03	enoyl CoA hydratase domain containing 3
2948	GSTM4	1.37	1.21E-02	glutathione S-transferase mu 4
2651	GCNT2	1.37	1.55E-02	glucosaminyl (N-acetyl) transferase 2, I-branching enzyme (I blood group)
81539	SLC38A1	1.37	1.83E-03	solute carrier family 38, member 1
5256	PHKA2	1.36	1.59E-03	phosphorylase kinase, alpha 2 (liver)
22853	LMTK2	1.36	4.74E-04	lemur tyrosine kinase 2
2052	EPHX1	1.36	2.78E-04	epoxide hydrolase 1, microsomal (xenobiotic)
79935	CNTD2	1.36	3.61E-02	cyclin N-terminal domain containing 2
27242	TNFRSF21	1.36	1.29E-02	tumor necrosis factor receptor superfamily, member 21
3625	INHBB	1.36	4.83E-02	inhibin, beta B
375757	SWI5	1.36	4.25E-03	SWI5 recombination repair homolog (yeast)
203228	C9orf72	1.36	3.67E-03	chromosome 9 open reading frame 72
3251	HPRT1	1.36	4.70E-04	hypoxanthine phosphoribosyltransferase 1
7873	MANF	1.36	1.02E-03	mesencephalic astrocyte-derived neurotrophic factor
100130742	LRRC69	1.36	2.85E-02	leucine rich repeat containing 69
493	ATP2B4	1.36	6.31E-03	ATPase, Ca++ transporting, plasma membrane 4
168451	THAP5	1.36	3.53E-02	THAP domain containing 5
9942	XYLB	1.36	5.23E-03	xylulokinase homolog (H. influenzae)
8878	SQSTM1	1.36	4.02E-05	sequestosome 1
3159	HMGA1	1.35	8.87E-03	high mobility group AT-hook 1
128853	DUSP15	1.35	1.74E-02	dual specificity phosphatase 15
27090	ST6GALNAC4	1.35	8.74E-04	ST6 (alpha-N-acetyl-neuraminyl-2,3-beta-galactosyl-1,3)-N-acetylgalactosaminide alpha-2,6-sialyltransferase 4
5009	OTC	1.35	3.01E-02	ornithine carbamoyltransferase
6520	SLC3A2	1.35	2.79E-04	solute carrier family 3 (amino acid transporter heavy chain), member 2
26064	RAI14	1.35	9.97E-04	retinoic acid induced 14
400798	C1orf220	1.35	1.05E-02	chromosome 1 open reading frame 220
54700	RRN3	1.35	2.47E-02	RRN3 RNA polymerase I transcription factor homolog (S. cerevisiae)
122970	ACOT4	1.35	3.11E-02	acyl-CoA thioesterase 4
84681	HINT2	1.35	1.56E-02	histidine triad nucleotide binding protein 2
6482	ST3GAL1	1.35	1.10E-03	ST3 beta-galactoside alpha-2,3-sialyltransferase 1
901	CCNG2	1.35	2.46E-02	cyclin G2
55728	N4BP2	1.35	1.81E-02	NEDD4 binding protein 2
8209	C21orf33	1.34	1.10E-03	chromosome 21 open reading frame 33
548645	DNAJC25	1.34	2.31E-03	DnaJ (Hsp40) homolog, subfamily C , member 25
6675	UAP1	1.34	3.83E-03	UDP-N-acteylglucosamine pyrophosphorylase 1
2274	FHL2	1.34	8.81E-03	four and a half LIM domains 2
91050	CCDC149	1.34	2.69E-02	coiled-coil domain containing 149
8309	ACOX2	1.34	3.84E-02	acyl-CoA oxidase 2, branched chain
645	BLVRB	1.34	1.52E-03	biliverdin reductase B (flavin reductase (NADPH))
55219	TMEM57	1.34	5.79E-03	transmembrane protein 57
7754	ZNF204P	1.34	3.85E-02	zinc finger protein 204, pseudogene
692099	FAM86DP	1.34	8.01E-04	family with sequence similarity 86, member D, pseudogene
10126	DNAL4	1.34	3.22E-02	dynein, axonemal, light chain 4
5243	ABCB1	1.34	9.46E-03	ATP-binding cassette, sub-family B (MDR/TAP), member 1
1119	CHKA	1.34	2.72E-02	choline kinase alpha
58510	PRODH2	1.34	1.24E-02	proline dehydrogenase (oxidase) 2
643246	MAP1LC3B2	1.34	4.21E-02	microtubule-associated protein 1 light chain 3 beta 2
9532	BAG2	1.34	1.94E-03	BCL2-associated athanogene 2
27440	CECR5	1.34	3.17E-02	cat eye syndrome chromosome region, candidate 5
4359	MPZ	1.34	2.74E-02	myelin protein zero
79739	TTLL7	1.33	2.93E-02	tubulin tyrosine ligase-like family, member 7
64816	CYP3A43	1.33	4.90E-02	cytochrome P450, family 3, subfamily A, polypeptide 43
271	AMPD2	1.33	7.37E-03	adenosine monophosphate deaminase 2
8027	STAM	1.33	1.78E-03	signal transducing adaptor molecule (SH3 domain and ITAM motif) 1
9955	HS3ST3A1	1.33	3.49E-02	heparan sulfate (glucosamine) 3-O-sulfotransferase 3A1
23753	SDF2L1	1.33	6.72E-03	stromal cell-derived factor 2-like 1
6786	STIM1	1.33	1.21E-03	stromal interaction molecule 1
135398	C6orf141	1.33	5.22E-03	chromosome 6 open reading frame 141
1373	CPS1	1.33	5.09E-03	carbamoyl-phosphate synthase 1, mitochondrial
26995	TRUB2	1.32	5.56E-04	TruB pseudouridine (psi) synthase family member 2
27230	SERP1	1.32	1.22E-03	stress-associated endoplasmic reticulum protein 1
8165	AKAP1	1.32	1.75E-03	A kinase (PRKA) anchor protein 1
256987	SERINC5	1.32	2.67E-03	serine incorporator 5
3290	HSD11B1	1.32	2.87E-03	hydroxysteroid (11-beta) dehydrogenase 1
189	AGXT	1.32	1.48E-03	alanine-glyoxylate aminotransferase
23743	BHMT2	1.32	4.30E-04	betaine--homocysteine S-methyltransferase 2
27347	STK39	1.32	3.73E-02	serine threonine kinase 39
171586	ABHD3	1.32	6.31E-03	abhydrolase domain containing 3
3081	HGD	1.32	2.11E-02	homogentisate 1,2-dioxygenase
8412	BCAR3	1.32	1.69E-02	breast cancer anti-estrogen resistance 3
79003	MIS12	1.32	1.07E-02	MIS12 kinetochore complex component
493856	CISD2	1.31	1.83E-02	CDGSH iron sulfur domain 2
10726	NUDC	1.31	1.28E-03	nudC nuclear distribution protein
84288	EFCAB2	1.31	3.61E-02	EF-hand calcium binding domain 2
55974	SLC50A1	1.31	7.27E-03	solute carrier family 50 (sugar efflux transporter), member 1
79701	OGFOD3	1.31	1.23E-02	2-oxoglutarate and iron-dependent oxygenase domain containing 3
114907	FBXO32	1.30	3.00E-02	F-box protein 32
399665	FAM102A	1.30	1.71E-02	family with sequence similarity 102, member A
1050	CEBPA	1.30	2.46E-02	CCAAT/enhancer binding protein (C/EBP), alpha
6836	SURF4	1.30	4.36E-04	surfeit 4
2878	GPX3	1.30	2.47E-03	glutathione peroxidase 3 (plasma)
51651	PTRH2	1.30	1.83E-02	peptidyl-tRNA hydrolase 2
84513	PPAPDC1B	1.30	6.39E-03	phosphatidic acid phosphatase type 2 domain containing 1B
7068	THRB	1.30	4.82E-02	thyroid hormone receptor, beta
202915	TMEM184A	1.30	1.54E-02	transmembrane protein 184A
84447	SYVN1	1.30	5.23E-03	synovial apoptosis inhibitor 1, synoviolin
8565	YARS	1.30	1.34E-03	tyrosyl-tRNA synthetase
2770	GNAI1	1.30	4.79E-02	guanine nucleotide binding protein (G protein), alpha inhibiting activity polypeptide 1
83752	LONP2	1.30	1.30E-03	lon peptidase 2, peroxisomal
56261	GPCPD1	1.30	4.59E-02	glycerophosphocholine phosphodiesterase GDE1 homolog (S. cerevisiae)
157922	CAMSAP1	1.30	5.74E-03	calmodulin regulated spectrin-associated protein 1
25791	NGEF	1.30	3.85E-02	neuronal guanine nucleotide exchange factor
219743	TYSND1	1.30	1.00E-02	trypsin domain containing 1
29968	PSAT1	1.30	3.16E-02	phosphoserine aminotransferase 1
286343	LURAP1L	1.29	2.66E-02	leucine rich adaptor protein 1-like
3775	KCNK1	1.29	2.11E-02	potassium channel, subfamily K, member 1
2035	EPB41	1.29	1.00E-02	erythrocyte membrane protein band 4.1 (elliptocytosis 1, RH-linked)
3655	ITGA6	1.29	2.42E-03	integrin, alpha 6

11162	NUDT6	1.29	3.01E-02	nudix (nucleoside diphosphate linked moiety X)-type motif 6
10165	SLC25A13	1.29	3.83E-03	solute carrier family 25 (aspartate/glutamate carrier), member 13
2717	GLA	1.29	1.78E-03	galactosidase, alpha
55343	SLC35C1	1.29	4.90E-02	solute carrier family 35 (GDP-fucose transporter), member C1
79161	TMEM243	1.29	3.00E-02	transmembrane protein 243, mitochondrial
2109	ETFB	1.29	5.58E-03	electron-transfer-flavoprotein, beta polypeptide
27304	MOCS3	1.29	4.82E-02	molybdenum cofactor synthesis 3
51136	RNFT1	1.29	1.17E-02	ring finger protein, transmembrane 1
114818	KLHL29	1.29	1.24E-02	kelch-like family member 29
3693	ITGB5	1.29	2.71E-03	integrin, beta 5
283464	GXYLT1	1.29	2.17E-03	glucoside xylosyltransferase 1
23576	DDAH1	1.29	2.09E-03	dimethylarginine dimethylaminohydrolase 1
79594	MUL1	1.29	1.48E-02	mitochondrial E3 ubiquitin protein ligase 1
1491	CTH	1.29	3.96E-02	cystathionase (cystathionine gamma-lyase)
11260	XPOT	1.28	9.69E-03	exportin, tRNA
7371	UCK2	1.28	6.56E-03	uridine-cytidine kinase 2
9821	RB1CC1	1.28	2.28E-03	RB1-inducible coiled-coil 1
196410	METTL7B	1.28	1.53E-02	methyltransferase like 7B
9697	TRAM2	1.28	2.19E-03	translocation associated membrane protein 2
200933	FBXO45	1.28	2.35E-02	F-box protein 45
3032	HADHB	1.28	1.95E-03	hydroxyacyl-CoA dehydrogenase/3-ketoacyl-CoA thiolase/enoyl-CoA hydratase (trifunctional protein), beta subunit
152641	WWC2-AS2	1.28	2.03E-02	WWC2 antisense RNA 2
9975	NR1D2	1.28	4.04E-03	nuclear receptor subfamily 1, group D, member 2
29958	DMGDH	1.28	3.89E-02	dimethylglycine dehydrogenase
57161	PELI2	1.28	1.03E-02	pellino E3 ubiquitin protein ligase family member 2
51099	ABHD5	1.28	3.68E-02	abhydrolase domain containing 5
8366	HIST1H4B	1.28	4.05E-02	histone cluster 1, H4b
10397	NDRG1	1.28	1.32E-02	N-myc downstream regulated 1
29925	GMPPB	1.28	1.33E-02	GDP-mannose pyrophosphorylase B
5795	PTPRJ	1.28	7.07E-03	protein tyrosine phosphatase, receptor type, J
85377	MICALL1	1.28	4.87E-02	MICAL-like 1
6249	CLIP1	1.28	9.72E-04	CAP-GLY domain containing linker protein 1
7922	SLC39A7	1.28	3.02E-03	solute carrier family 39 (zinc transporter), member 7
9093	DNAJA3	1.28	2.42E-03	DnaJ (Hsp40) homolog, subfamily A, member 3
80218	NAA50	1.28	3.30E-04	N(alpha)-acetyltransferase 50, NatE catalytic subunit
55605	KIF21A	1.28	4.19E-03	kinesin family member 21A
25850	ZNF345	1.28	4.54E-02	zinc finger protein 345
84725	PLEKHA8	1.28	3.54E-02	pleckstrin homology domain containing, family A (phosphoinositide binding specific) member 8
55435	AP1AR	1.28	4.96E-03	adaptor-related protein complex 1 associated regulatory protein
10560	SLC19A2	1.27	3.17E-02	solute carrier family 19 (thiamine transporter), member 2
84818	IL17RC	1.27	2.95E-02	interleukin 17 receptor C
23277	CLUH	1.27	1.55E-03	clustered mitochondria (cluA/CLU1) homolog
216	ALDH1A1	1.27	8.23E-03	aldehyde dehydrogenase 1 family, member A1
5053	PAH	1.27	4.16E-02	phenylalanine hydroxylase
133	ADM	1.27	2.75E-02	adrenomedullin
28955	DEXI	1.27	3.71E-02	Dexi homolog (mouse)
91526	ANKRD44	1.27	8.26E-03	ankyrin repeat domain 44
9980	DOPEY2	1.27	2.80E-02	dopey family member 2
2247	FGF2	1.27	1.70E-02	fibroblast growth factor 2 (basic)
83606	GUCD1	1.27	8.74E-04	guanylyl cyclase domain containing 1
64755	C16orf58	1.27	8.02E-03	chromosome 16 open reading frame 58
2646	GCKR	1.27	2.82E-02	glucokinase (hexokinase 4) regulator
5062	PAK2	1.27	8.74E-04	p21 protein (Cdc42/Rac)-activated kinase 2
1843	DUSP1	1.27	4.50E-04	dual specificity phosphatase 1
5217	PFN2	1.27	4.91E-03	profilin 2
51114	ZDHHC9	1.27	2.55E-03	zinc finger, DHHC-type containing 9
64216	TFB2M	1.27	7.34E-03	transcription factor B2, mitochondrial
201232	SLC16A13	1.27	1.42E-02	solute carrier family 16, member 13
57414	RHBDD2	1.27	7.75E-03	rhomboid domain containing 2
56288	PARD3	1.27	7.37E-04	par-3 family cell polarity regulator
3741	KCNA5	1.27	4.52E-02	potassium voltage-gated channel, shaker-related subfamily, member 5
2872	MKNK2	1.27	3.98E-02	MAP kinase interacting serine/threonine kinase 2
10483	SEC23B	1.26	3.44E-03	Sec23 homolog B (S. cerevisiae)
56997	ADCK3	1.26	4.95E-02	aarF domain containing kinase 3
121260	SLC15A4	1.26	1.01E-02	solute carrier family 15 (oligopeptide transporter), member 4
22928	SEPHS2	1.26	3.48E-03	selenophosphate synthetase 2
5387	PMS2P3	1.26	1.24E-02	postmeiotic segregation increased 2 pseudogene 3
4953	ODC1	1.26	1.79E-03	ornithine decarboxylase 1
57834	CYP4F11	1.26	2.04E-02	cytochrome P450, family 4, subfamily F, polypeptide 11
5621	PRNP	1.26	3.26E-03	prion protein
29088	MRPL15	1.26	4.38E-02	mitochondrial ribosomal protein L15
79144	PPDPF	1.26	1.78E-02	pancreatic progenitor cell differentiation and proliferation factor
29926	GMPPA	1.26	8.97E-03	GDP-mannose pyrophosphorylase A
23223	RRP12	1.26	4.19E-03	ribosomal RNA processing 12 homolog (S. cerevisiae)
55330	BLOC1S4	1.26	4.91E-02	biogenesis of lysosomal organelles complex-1, subunit 4, cappuccino
10525	HYOU1	1.26	1.19E-02	hypoxia up-regulated 1
1978	EIF4EBP1	1.26	2.50E-02	eukaryotic translation initiation factor 4E binding protein 1
1579	CYP4A11	1.26	3.27E-02	cytochrome P450, family 4, subfamily A, polypeptide 11
51058	ZNF691	1.26	4.33E-02	zinc finger protein 691
54929	TMEM161A	1.26	4.43E-02	transmembrane protein 161A
63893	UBE2O	1.26	1.42E-02	ubiquitin-conjugating enzyme E2O
10400	PEMT	1.26	1.11E-02	phosphatidylethanolamine N-methyltransferase
6782	HSPA13	1.26	2.76E-03	heat shock protein 70kDa family, member 13
22809	ATF5	1.26	1.18E-02	activating transcription factor 5
254887	ZDHHC23	1.26	1.05E-02	zinc finger, DHHC-type containing 23
9570	GOSR2	1.26	6.56E-03	golgi SNAP receptor complex member 2
84649	DGAT2	1.25	3.46E-02	diacylglycerol O-acyltransferase 2
79875	THSD4	1.25	3.73E-02	thrombospondin, type I, domain containing 4
55884	WSB2	1.25	4.75E-03	WD repeat and SOCS box containing 2
7923	HSD17B8	1.25	9.05E-03	hydroxysteroid (17-beta) dehydrogenase 8
134147	CMBL	1.25	1.03E-02	carboxymethylenebutenolidase homolog (Pseudomonas)
8608	RDH16	1.25	3.60E-02	retinol dehydrogenase 16 (all-trans)
2669	GEM	1.25	1.83E-02	GTP binding protein overexpressed in skeletal muscle
144811	LACC1	1.25	1.29E-02	laccase (multicopper oxidoreductase) domain containing 1
221756	SERPINB9P1	1.25	4.95E-02	serpin peptidase inhibitor, clade B (ovalbumin), member 9, pseudogene 1
7132	TNFRSF1A	1.25	2.76E-03	tumor necrosis factor receptor superfamily, member 1A
8439	NSMAF	1.25	1.97E-02	neutral sphingomyelinase (N-SMase) activation associated factor
55132	LARP1B	1.25	3.17E-02	La ribonucleoprotein domain family, member 1B
9429	ABCG2	1.25	3.67E-02	ATP-binding cassette, sub-family G (WHITE), member 2
200844	C3orf67	1.25	2.98E-02	chromosome 3 open reading frame 67
29914	UBIAD1	1.25	1.47E-02	UbiA prenyltransferase domain containing 1

56947	MFF	1.24	9.39E-03	mitochondrial fission factor
9689	BZW1	1.24	1.29E-02	basic leucine zipper and W2 domains 1
10099	TSPAN3	1.24	3.96E-03	tetraspanin 3
10933	MORF4L1	1.24	1.62E-02	mortality factor 4 like 1
8187	ZNF239	1.24	4.52E-02	zinc finger protein 239
30836	DNTTIP2	1.24	7.07E-03	deoxynucleotidyltransferase, terminal, interacting protein 2
5565	PRKAB2	1.24	2.10E-02	protein kinase, AMP-activated, beta 2 non-catalytic subunit
84085	FBXO30	1.24	1.77E-03	F-box protein 30
5830	PEX5	1.24	7.83E-03	peroxisomal biogenesis factor 5
54867	TMEM214	1.24	1.66E-02	transmembrane protein 214
347735	SERINC2	1.24	1.54E-02	serine incorporator 2
5164	PDK2	1.24	3.99E-02	pyruvate dehydrogenase kinase, isozyme 2
26777	SNORA71A	1.24	2.47E-02	small nucleolar RNA, H/ACA box 71A
23483	TGDS	1.24	8.81E-03	TDP-glucose 4,6-dehydratase
7184	HSP90B1	1.24	1.56E-03	heat shock protein 90kDa beta (Grp94), member 1
60314	C12orf10	1.24	1.55E-02	chromosome 12 open reading frame 10
51582	AZIN1	1.23	7.57E-04	antizyme inhibitor 1
221656	KDM1B	1.23	1.34E-02	lysine (K)-specific demethylase 1B
84336	TMEM101	1.23	4.08E-02	transmembrane protein 101
10613	ERLIN1	1.23	2.22E-02	ER lipid raft associated 1
3998	LMAN1	1.23	1.55E-02	lectin, mannose-binding, 1
162989	DEDD2	1.23	4.10E-02	death effector domain containing 2
23139	MAST2	1.23	8.15E-03	microtubule associated serine/threonine kinase 2
10598	AHSA1	1.23	1.84E-03	AHA1, activator of heat shock 90kDa protein ATPase homolog 1 (yeast)
64795	RMND5A	1.23	4.95E-02	required for meiotic nuclear division 5 homolog A (S. cerevisiae)
64425	POLR1E	1.23	2.08E-02	polymerase (RNA) I polypeptide E, 53kDa
54676	GTPBP2	1.23	2.92E-02	GTP binding protein 2
153241	CEP120	1.23	4.82E-02	centrosomal protein 120kDa
360023	ZBTB41	1.23	3.01E-02	zinc finger and BTB domain containing 41
63971	KIF13A	1.23	1.16E-02	kinesin family member 13A
79018	GID4	1.23	4.71E-02	GID complex subunit 4
55313	CPPED1	1.23	2.45E-03	calcineurin-like phosphoesterase domain containing 1
58477	SRPRB	1.23	2.54E-03	signal recognition particle receptor, B subunit
51649	MRPS23	1.23	3.70E-02	mitochondrial ribosomal protein S23
7514	XPO1	1.23	7.72E-03	exportin 1
64175	LEPRE1	1.23	3.41E-02	leucine proline-enriched proteoglycan (leprecan) 1
978	CDA	1.22	1.86E-02	cytidine deaminase
11108	PRDM4	1.22	1.28E-02	PR domain containing 4
7071	KLF10	1.22	5.44E-03	Kruppel-like factor 10
96610	BMS1P20	1.22	2.96E-02	BMS1 pseudogene 20
90411	MCFD2	1.22	6.31E-03	multiple coagulation factor deficiency 2
7165	TPD52L2	1.22	4.91E-03	tumor protein D52-like 2
3030	HADHA	1.22	1.70E-02	hydroxyacyl-CoA dehydrogenase/3-ketoacyl-CoA thiolase/enoyl-CoA hydratase (trifunctional protein), alpha subunit
5167	ENPP1	1.22	1.11E-02	ectonucleotide pyrophosphatase/phosphodiesterase 1
50640	PNPLA8	1.22	2.19E-02	patatin-like phospholipase domain containing 8
64771	C6orf106	1.22	8.02E-03	chromosome 6 open reading frame 106
375035	SFT2D2	1.22	1.94E-02	SFT2 domain containing 2
26207	PITPNC1	1.22	9.59E-03	phosphatidylinositol transfer protein, cytoplasmic 1
26151	NAT9	1.22	2.75E-02	N-acetyltransferase 9 (GCN5-related, putative)
5412	UBL3	1.22	9.85E-03	ubiquitin-like 3
27248	ERLEC1	1.22	2.49E-02	endoplasmic reticulum lectin 1
91252	SLC39A13	1.22	2.42E-02	solute carrier family 39 (zinc transporter), member 13
9557	CHD1L	1.22	3.63E-02	chromodomain helicase DNA binding protein 1-like
54469	ZFAND6	1.22	3.09E-02	zinc finger, AN1-type domain 6
10196	PRMT3	1.22	2.69E-02	protein arginine methyltransferase 3
80273	GRPEL1	1.22	7.67E-03	GrpE-like 1, mitochondrial (E. coli)
10838	ZNF275	1.22	2.47E-02	zinc finger protein 275
51566	ARMCX3	1.22	3.95E-02	armadillo repeat containing, X-linked 3
26608	TBL2	1.22	4.81E-02	transducin (beta)-like 2
9917	FAM20B	1.21	1.10E-02	family with sequence similarity 20, member B
7419	VDAC3	1.21	5.82E-04	voltage-dependent anion channel 3
54517	PUS7	1.21	1.76E-02	pseudouridylate synthase 7 (putative)
3295	HSD17B4	1.21	1.41E-02	hydroxysteroid (17-beta) dehydrogenase 4
400	ARL1	1.21	2.02E-02	ADP-ribosylation factor-like 1
8907	AP1M1	1.21	3.82E-02	adaptor-related protein complex 1, mu 1 subunit
5054	SERPINE1	1.21	2.61E-02	serpin peptidase inhibitor, clade E (nexin, plasminogen activator inhibitor type 1), member 1
8100	IFT88	1.21	2.46E-02	intraflagellar transport 88 homolog (Chlamydomonas)
85379	KIAA1671	1.21	1.10E-02	KIAA1671
2801	GOLGA2	1.21	1.67E-02	golgin A2
10322	SMYD5	1.21	2.74E-02	SMYD family member 5
4780	NFE2L2	1.21	1.39E-02	nuclear factor, erythroid 2-like 2
84919	PPP1R15B	1.21	2.14E-02	protein phosphatase 1, regulatory subunit 15B
57222	ERGIC1	1.21	4.17E-03	endoplasmic reticulum-golgi intermediate compartment (ERGIC) 1
27075	TSPAN13	1.20	3.73E-02	tetraspanin 13
4199	ME1	1.20	1.04E-02	malic enzyme 1, NADP(+)-dependent, cytosolic
91272	BOD1	1.20	1.49E-02	biorientation of chromosomes in cell division 1
5467	PPARD	1.20	3.13E-02	peroxisome proliferator-activated receptor delta
9146	HGS	1.20	1.70E-02	hepatocyte growth factor-regulated tyrosine kinase substrate
56647	BCCIP	1.20	3.36E-02	BRCA2 and CDKN1A interacting protein
1979	EIF4EBP2	1.20	2.57E-03	eukaryotic translation initiation factor 4E binding protein 2
56683	C21orf59	1.20	1.20E-02	chromosome 21 open reading frame 59
6746	SSR2	1.20	1.11E-02	signal sequence receptor, beta (translocon-associated protein beta)
4233	MET	1.20	3.45E-02	met proto-oncogene
7965	AIMP2	1.20	1.70E-02	aminoacyl tRNA synthetase complex-interacting multifunctional protein 2
6683	SPAST	1.20	1.56E-02	spastin
1317	SLC31A1	1.20	2.19E-02	solute carrier family 31 (copper transporter), member 1
7295	TXN	1.20	1.00E-02	thioredoxin
6780	STAU1	1.20	3.62E-03	staufen double-stranded RNA binding protein 1
134553	C5orf24	1.20	3.17E-02	chromosome 5 open reading frame 24
81671	VMP1	1.20	1.94E-03	vacuole membrane protein 1
56910	STARD7	1.20	3.38E-03	StAR-related lipid transfer (START) domain containing 7
1497	CTNS	1.20	2.93E-02	cystinosin, lysosomal cystine transporter
5686	PSMA5	1.20	3.64E-02	proteasome (prosome, macropain) subunit, alpha type, 5
55829	VIMP	1.20	3.25E-02	VCP-interacting membrane protein
11046	SLC35D2	1.20	2.81E-02	solute carrier family 35 (UDP-GlcNAc/UDP-glucose transporter), member D2
10455	ECI2	1.19	9.14E-03	enoyl-CoA delta isomerase 2
6732	SRPK1	1.19	2.45E-02	SRSF protein kinase 1
7764	ZNF217	1.19	3.37E-02	zinc finger protein 217
51061	TXNDC11	1.19	3.67E-02	thioredoxin domain containing 11
8772	FADD	1.19	3.69E-02	Fas (TNFRSF6)-associated via death domain
10953	TOMM34	1.19	1.34E-02	translocase of outer mitochondrial membrane 34

63929	XPNPEP3	1.19	4.99E-02	X-prolyl aminopeptidase (aminopeptidase P) 3, putative
10130	PDIA6	1.19	2.18E-02	protein disulfide isomerase family A, member 6
29855	UBN1	1.19	4.92E-02	ubinuclein 1
7813	EVI5	1.19	3.70E-02	ecotropic viral integration site 5
10728	PTGES3	1.19	1.75E-02	prostaglandin E synthase 3 (cytosolic)
9184	BUB3	1.19	3.57E-02	BUB3 mitotic checkpoint protein
10120	ACTR1B	1.19	3.47E-02	ARP1 actin-related protein 1 homolog B, centractin beta (yeast)
4257	MGST1	1.19	5.78E-03	microsomal glutathione S-transferase 1
613	BCR	1.19	4.16E-02	breakpoint cluster region
8630	HSD17B6	1.19	2.10E-02	hydroxysteroid (17-beta) dehydrogenase 6
23230	VPS13A	1.19	2.88E-02	vacuolar protein sorting 13 homolog A (S. cerevisiae)
26354	GNL3	1.19	1.40E-02	guanine nucleotide binding protein-like 3 (nucleolar)
10885	WDR3	1.19	2.75E-02	WD repeat domain 3
25923	ATL3	1.19	6.77E-03	atlastin GTPase 3
84522	JAGN1	1.19	3.31E-02	jagunal homolog 1 (Drosophila)
25888	ZNF473	1.19	3.55E-02	zinc finger protein 473
55907	CMAS	1.19	4.84E-02	cytidine monophosphate N-acetylneuraminic acid synthetase
127262	TPRG1L	1.19	4.14E-02	tumor protein p63 regulated 1-like
22931	RAB18	1.18	8.02E-03	RAB18, member RAS oncogene family
85021	REPS1	1.18	5.26E-03	RALBP1 associated Eps domain containing 1
9950	GOLGA5	1.18	4.72E-02	golgin A5
142891	SAMD8	1.18	1.56E-02	sterile alpha motif domain containing 8
8886	DDX18	1.18	1.29E-02	DEAD (Asp-Glu-Ala-Asp) box polypeptide 18
58513	EPS15L1	1.18	3.86E-02	epidermal growth factor receptor pathway substrate 15-like 1
7342	UBP1	1.18	2.89E-02	upstream binding protein 1 (LBP-1a)
7267	TTC3	1.18	3.24E-02	tetratricopeptide repeat domain 3
50865	HEBP1	1.18	4.12E-02	heme binding protein 1
51430	SUCO	1.18	2.47E-02	SUN domain containing ossification factor
6868	ADAM17	1.18	3.15E-02	ADAM metallopeptidase domain 17
116064	LRRC58	1.18	3.71E-02	leucine rich repeat containing 58
9921	RNF10	1.18	1.38E-02	ring finger protein 10
9043	SPAG9	1.18	4.48E-02	sperm associated antigen 9
22937	SCAP	1.18	2.98E-02	SREBF chaperone
92140	MTDH	1.18	6.72E-03	metadherin
30	ACAA1	1.18	1.15E-02	acetyl-CoA acyltransferase 1
8882	ZPR1	1.18	2.36E-02	ZPR1 zinc finger
57587	KIAA1430	1.18	1.97E-02	KIAA1430
200734	SPRED2	1.18	3.34E-02	sprouty-related, EVH1 domain containing 2
8476	CDC42BPA	1.18	3.17E-02	CDC42 binding protein kinase alpha (DMPK-like)
55760	DHX32	1.17	1.49E-02	DEAH (Asp-Glu-Ala-His) box polypeptide 32
55003	PAK1IP1	1.17	1.97E-02	PAK1 interacting protein 1
2288	FKBP4	1.17	2.65E-02	FK506 binding protein 4, 59kDa
262	AMD1	1.17	2.30E-02	adenosylmethionine decarboxylase 1
80854	SETD7	1.17	7.61E-03	SET domain containing (lysine methyltransferase) 7
7110	TMF1	1.17	1.86E-02	TATA element modulatory factor 1
51012	SLMO2	1.17	2.96E-02	slowmo homolog 2 (Drosophila)
10105	PPIF	1.17	2.82E-02	peptidylprolyl isomerase F
84246	MED10	1.17	2.83E-02	mediator complex subunit 10
3633	INPP5B	1.17	4.68E-02	inositol polyphosphate-5-phosphatase, 75kDa
169200	TMEM64	1.17	2.18E-02	transmembrane protein 64
85403	EAF1	1.17	1.70E-02	ELL associated factor 1
9731	CEP104	1.17	3.01E-02	centrosomal protein 104kDa
7106	TSPAN4	1.17	2.74E-02	tetraspanin 4
9475	ROCK2	1.17	1.93E-02	Rho-associated, coiled-coil containing protein kinase 2
55127	HEATR1	1.17	4.95E-02	HEAT repeat containing 1
55667	DENND4C	1.17	2.81E-02	DENN/MADD domain containing 4C
58472	SQRDL	1.17	4.00E-02	sulfide quinone reductase-like (yeast)
5929	RBBP5	1.17	4.78E-02	retinoblastoma binding protein 5
22870	PPP6R1	1.17	3.57E-02	protein phosphatase 6, regulatory subunit 1
3459	IFNGR1	1.17	2.76E-03	interferon gamma receptor 1
9654	TTLL4	1.17	3.20E-02	tubulin tyrosine ligase-like family, member 4
1604	CD55	1.17	3.17E-02	CD55 molecule, decay accelerating factor for complement (Cromer blood group)
3692	EIF6	1.17	5.00E-02	eukaryotic translation initiation factor 6
5352	PLOD2	1.16	4.45E-02	procollagen-lysine, 2-oxoglutarate 5-dioxygenase 2
4170	MCL1	1.16	1.08E-02	myeloid cell leukemia 1
26003	GORASP2	1.16	1.54E-02	golgi reassembly stacking protein 2, 55kDa
2317	FLNB	1.16	2.14E-02	filamin B, beta
4927	NUP88	1.16	3.98E-02	nucleoporin 88kDa
2803	GOLGA4	1.16	8.74E-03	golgin A4
155061	ZNF746	1.16	3.54E-02	zinc finger protein 746
55054	ATG16L1	1.16	4.94E-02	autophagy related 16-like 1 (S. cerevisiae)
1983	EIF5	1.16	2.47E-02	eukaryotic translation initiation factor 5
26100	WIPI2	1.16	4.90E-02	WD repeat domain, phosphoinositide interacting 2
5092	PCBD1	1.16	2.95E-02	pterin-4 alpha-carbinolamine dehydratase/dimerization cofactor of hepatocyte nuclear factor 1 alpha
5203	PFDN4	1.16	4.65E-02	prefoldin subunit 4
51181	DCXR	1.16	4.12E-02	dicarbonyl/L-xylulose reductase
571	BACH1	1.16	4.56E-02	BTB and CNC homology 1, basic leucine zipper transcription factor 1
3312	HSPA8	1.16	9.45E-03	heat shock 70kDa protein 8
4799	NFX1	1.16	3.95E-02	nuclear transcription factor, X-box binding 1
79691	QTRTD1	1.16	4.75E-02	queuine tRNA-ribosyltransferase domain containing 1
3376	IARS	1.16	2.42E-02	isoleucyl-tRNA synthetase
3313	HSPA9	1.16	1.31E-02	heat shock 70kDa protein 9 (mortalin)
6856	SYPL1	1.16	3.57E-02	synaptophysin-like 1
27339	PRPF19	1.15	4.77E-02	pre-mRNA processing factor 19
8615	USO1	1.15	2.81E-02	USO1 vesicle transport factor
221749	PXDC1	1.15	3.71E-02	PX domain containing 1
5264	PHYH	1.15	3.01E-02	phytanoyl-CoA 2-hydroxylase
1650	DDOST	1.15	4.85E-02	dolichyl-diphosphooligosaccharide--protein glycosyltransferase subunit (non-catalytic)
1212	CLTB	1.15	3.72E-02	clathrin, light chain B
10969	EBNA1BP2	1.15	2.73E-02	EBNA1 binding protein 2
10857	PGRMC1	1.15	6.31E-03	progesterone receptor membrane component 1
8833	GMPS	1.15	4.25E-02	guanine monphosphate synthase
8454	CUL1	1.15	1.03E-02	cullin 1
60481	ELOVL5	1.15	1.40E-02	ELOVL fatty acid elongase 5
201627	DENND6A	1.14	3.19E-02	DENN/MADD domain containing 6A
7415	VCP	1.14	4.53E-03	valosin containing protein
23214	XPO6	1.14	2.63E-02	exportin 6
7763	ZFAND5	1.14	1.48E-02	zinc finger, AN1-type domain 5
7358	UGDH	1.14	8.38E-03	UDP-glucose 6-dehydrogenase
5007	OSBP	1.14	4.78E-02	oxysterol binding protein
3069	HDLBP	1.14	4.45E-02	high density lipoprotein binding protein

23074	UHRF1BP1L	1.14	3.84E-02	UHRF1 binding protein 1-like
3326	HSP90AB1	1.14	1.56E-02	heat shock protein 90kDa alpha (cytosolic), class B member 1
92856	IMP4	1.14	1.33E-02	IMP4, U3 small nucleolar ribonucleoprotein
55970	GNG12	1.13	2.98E-02	guanine nucleotide binding protein (G protein), gamma 12
9275	BCL7B	1.13	3.34E-02	B-cell CLL/lymphoma 7B
4259	MGST3	1.13	4.79E-02	microsomal glutathione S-transferase 3
1635	DCTD	1.13	3.73E-02	dCMP deaminase
3416	IDE	1.13	3.17E-02	insulin-degrading enzyme
94056	SYAP1	1.13	2.35E-02	synapse associated protein 1
821	CANX	1.12	2.87E-02	calnexin
3267	AGFG1	1.12	3.58E-02	ArfGAP with FG repeats 1
5052	PRDX1	1.11	4.10E-02	peroxiredoxin 1
