# Supplementary material for: The impact of PPARα activation on whole genome gene expression in human precision cut liver slices
Source: BMC Genomics. 2015 Oct 8;16:760. doi: 10.1186/s12864-015-1969-3 (PMC4599789; doi:10.1186/s12864-015-1969-3)
Supplement: Additional file 2: — Full list of significantly repressed genes by Wy14643 in human PCLS. (PDF 228 kb) [file 12864_2015_1969_MOESM2_ESM.pdf]

Entrez ID	gene name	fold change	q-value	description
4283	CXCL9	-7.25	5.28E-05	chemokine (C-X-C motif) ligand 9
3627	CXCL10	-6.88	6.58E-05	chemokine (C-X-C motif) ligand 10
6373	CXCL11	-5.65	3.69E-04	chemokine (C-X-C motif) ligand 11
405753	DUOXA2	-3.97	3.05E-06	dual oxidase maturation factor 2
4843	NOS2	-3.62	5.43E-03	nitric oxide synthase 2, inducible
50506	DUOX2	-3.24	5.01E-06	dual oxidase 2
6355	CCL8	-3.07	3.67E-03	chemokine (C-C motif) ligand 8
10964	IFI44L	-3.06	4.43E-04	interferon-induced protein 44-like
115362	GBP5	-2.94	6.83E-04	guanylate binding protein 5
3620	IDO1	-2.91	5.65E-06	indoleamine 2,3-dioxygenase 1
8519	IFITM1	-2.67	5.65E-06	interferon induced transmembrane protein 1
3433	IFIT2	-2.61	2.28E-03	interferon-induced protein with tetratricopeptide repeats 2
54898	ELOVL2	-2.61	4.38E-07	ELOVL fatty acid elongase 2
2892	GRIA3	-2.60	3.06E-05	glutamate receptor, ionotropic, AMPA 3
6376	CX3CL1	-2.57	4.43E-04	chemokine (C-X3-C motif) ligand 1
7098	TLR3	-2.55	5.76E-06	toll-like receptor 3
79689	STEAP4	-2.50	8.35E-05	STEAP family member 4
3434	IFIT1	-2.48	2.64E-03	interferon-induced protein with tetratricopeptide repeats 1
4321	MMP12	-2.45	2.30E-04	matrix metallopeptidase 12 (macrophage elastase)
10826	FAXDC2	-2.42	5.01E-06	fatty acid hydroxylase domain containing 2
8626	TP63	-2.41	2.02E-05	tumor protein p63
64577	ALDH8A1	-2.41	6.05E-06	aldehyde dehydrogenase 8 family, member A1
8740	TNFSF14	-2.40	6.35E-05	tumor necrosis factor (ligand) superfamily, member 14
10417	SPON2	-2.39	2.46E-06	spondin 2, extracellular matrix protein
3437	IFIT3	-2.38	2.74E-04	interferon-induced protein with tetratricopeptide repeats 3
54739	XAF1	-2.35	3.46E-06	XIAP associated factor 1
94240	EPSTI1	-2.29	2.79E-04	epithelial stromal interaction 1 (breast)
10346	TRIM22	-2.28	4.50E-06	tripartite motif containing 22
83953	FCAMR	-2.28	1.16E-02	Fc receptor, IgA, IgM, high affinity
3934	LCN2	-2.26	1.95E-04	lipocalin 2
388646	GBP7	-2.25	1.82E-04	guanylate binding protein 7
57817	HAMP	-2.24	5.28E-05	hepcidin antimicrobial peptide
2634	GBP2	-2.22	6.65E-05	guanylate binding protein 2, interferon-inducible
9288	TAAR3	-2.22	2.65E-02	trace amine associated receptor 3 (gene/pseudogene)
115361	GBP4	-2.21	2.38E-04	guanylate binding protein 4
6372	CXCL6	-2.20	8.74E-04	chemokine (C-X-C motif) ligand 6
10537	UBD	-2.19	1.02E-06	ubiquitin D
11274	USP18	-2.18	3.82E-04	ubiquitin specific peptidase 18
51156	SERPINA10	-2.18	2.15E-05	serpin peptidase inhibitor, clade A (alpha-1 antiproteinase, antitrypsin), member 10
6999	TDO2	-2.16	9.97E-03	tryptophan 2,3-dioxygenase
256764	WDR72	-2.15	1.02E-03	WD repeat domain 72
5920	RARRES3	-2.12	1.34E-06	retinoic acid receptor responder (tazarotene induced) 3
5284	PIGR	-2.11	1.97E-03	polymeric immunoglobulin receptor
4939	OAS2	-2.10	5.36E-04	2'-5'-oligoadenylate synthetase 2, 69/71kDa
113146	AHNAK2	-2.09	6.05E-06	AHNAK nucleoprotein 2
4599	MX1	-2.07	5.70E-04	myxovirus (influenza virus) resistance 1, interferon-inducible protein p78 (mouse)
4940	OAS3	-2.06	4.49E-04	2'-5'-oligoadenylate synthetase 3, 100kDa
57507	ZNF608	-2.06	5.01E-06	zinc finger protein 608
2633	GBP1	-2.04	1.05E-04	guanylate binding protein 1, interferon-inducible
3626	INHBC	-2.03	9.40E-05	inhibin, beta C
117153	MIA2	-2.02	2.07E-04	melanoma inhibitory activity 2
80833	APOL3	-2.01	1.86E-04	apolipoprotein L, 3
10068	IL18BP	-1.97	4.50E-06	interleukin 18 binding protein
54809	SAMD9	-1.97	2.61E-05	sterile alpha motif domain containing 9
84873	GPR128	-1.97	4.02E-05	G protein-coupled receptor 128
5168	ENPP2	-1.97	4.50E-06	ectonucleotide pyrophosphatase/phosphodiesterase 2
5207	PFKFB1	-1.96	3.69E-04	6-phosphofructo-2-kinase/fructose-2,6-biphosphatase 1
10272	FSTL3	-1.96	6.80E-03	follistatin-like 3 (secreted glycoprotein)
6289	SAA2	-1.96	1.46E-03	serum amyloid A2
10561	IFI44	-1.95	2.40E-04	interferon-induced protein 44
420	ART4	-1.94	1.30E-04	ADP-ribosyltransferase 4 (Dombrock blood group)
219285	SAMD9L	-1.94	7.48E-04	sterile alpha motif domain containing 9-like
4938	OAS1	-1.94	1.86E-04	2'-5'-oligoadenylate synthetase 1, 40/46kDa
80380	PDCD1LG2	-1.93	1.30E-03	programmed cell death 1 ligand 2
23586	DDX58	-1.93	8.22E-05	DEAD (Asp-Glu-Ala-Asp) box polypeptide 58
9636	ISG15	-1.92	8.26E-04	ISG15 ubiquitin-like modifier
4547	MTTP	-1.91	1.65E-04	microsomal triglyceride transfer protein
343450	KCNT2	-1.90	6.93E-05	potassium channel, subfamily T, member 2
5288	PIK3C2G	-1.90	2.58E-04	phosphatidylinositol-4-phosphate 3-kinase, catalytic subunit type 2 gamma
2537	IFI6	-1.89	1.29E-05	interferon, alpha-inducible protein 6
1592	CYP26A1	-1.89	7.57E-04	cytochrome P450, family 26, subfamily A, polypeptide 1
342372	PKD1L3	-1.89	2.15E-03	polycystic kidney disease 1-like 3
353514	LILRA5	-1.87	1.46E-03	leukocyte immunoglobulin-like receptor, subfamily A (with TM domain), member 5
7042	TGFB2	-1.87	2.30E-06	transforming growth factor, beta 2
23767	FLRT3	-1.86	2.25E-04	fibronectin leucine rich transmembrane protein 3
83666	PARP9	-1.86	3.18E-05	poly (ADP-ribose) polymerase family, member 9
412	STS	-1.85	4.62E-04	steroid sulfatase (microsomal), isozyme S
10677	AVIL	-1.84	4.43E-04	advillin
83729	INHBE	-1.84	6.47E-04	inhibin, beta E
163351	GBP6	-1.82	1.65E-02	guanylate binding protein family, member 6
2635	GBP3	-1.82	3.43E-04	guanylate binding protein 3
55008	HERC6	-1.81	3.76E-03	HECT and RLD domain containing E3 ubiquitin protein ligase family member 6
6274	S100A3	-1.81	4.79E-02	S100 calcium binding protein A3
55601	DDX60	-1.81	3.68E-04	DEAD (Asp-Glu-Ala-Asp) box polypeptide 60
114899	C1QTNF3	-1.80	2.75E-02	C1q and tumor necrosis factor related protein 3
27289	RND1	-1.79	1.56E-04	Rho family GTPase 1
952	CD38	-1.78	6.84E-04	CD38 molecule

9510	ADAMTS1	-1.78	2.41E-02	ADAM metallopeptidase with thrombospondin type 1 motif, 1
5737	PTGFR	-1.78	7.03E-04	prostaglandin F receptor (FP)
51056	LAP3	-1.78	1.46E-03	leucine aminopeptidase 3
64108	RTP4	-1.77	1.33E-03	receptor (chemosensory) transporter protein 4
6363	CCL19	-1.77	5.33E-05	chemokine (C-C motif) ligand 19
4068	SH2D1A	-1.77	1.20E-02	SH2 domain containing 1A
7127	TNFAIP2	-1.76	5.43E-03	tumor necrosis factor, alpha-induced protein 2
28951	TRIB2	-1.75	1.94E-03	tribbles pseudokinase 2
222643	UNC5CL	-1.75	1.02E-03	unc-5 homolog C (C. elegans)-like
9076	CLDN1	-1.75	1.06E-06	claudin 1
6690	SPINK1	-1.74	3.81E-03	serine peptidase inhibitor, Kazal type 1
200373	PCDP1	-1.74	1.56E-03	primary ciliary dyskinesia protein 1
6352	CCL5	-1.74	6.93E-04	chemokine (C-C motif) ligand 5
8875	VNN2	-1.73	7.42E-04	vanin 2
11074	TRIM31	-1.73	6.09E-05	tripartite motif containing 31
8743	TNFSF10	-1.73	1.99E-06	tumor necrosis factor (ligand) superfamily, member 10
118932	ANKRD22	-1.73	8.29E-03	ankyrin repeat domain 22
3430	IFI35	-1.72	5.43E-03	interferon-induced protein 35
3560	IL2RB	-1.72	2.29E-03	interleukin 2 receptor, beta
8470	SORBS2	-1.72	9.72E-04	sorbin and SH3 domain containing 2
60489	APOBEC3G	-1.72	2.10E-02	apolipoprotein B mRNA editing enzyme, catalytic polypeptide-like 3G
5698	PSMB9	-1.72	1.20E-03	proteasome (prosome, macropain) subunit, beta type, 9
7045	TGFBI	-1.71	1.04E-04	transforming growth factor, beta-induced, 68kDa
2877	GPX2	-1.71	8.74E-03	glutathione peroxidase 2 (gastrointestinal)
51513	ETV7	-1.71	1.86E-02	ets variant 7
284111	SLC13A5	-1.71	4.83E-04	solute carrier family 13 (sodium-dependent citrate transporter), member 5
6288	SAA1	-1.71	3.91E-04	serum amyloid A1
5320	PLA2G2A	-1.70	1.06E-03	phospholipase A2, group IIA (platelets, synovial fluid)
4212	MEIS2	-1.70	2.58E-04	Meis homeobox 2
64127	NOD2	-1.70	2.34E-02	nucleotide-binding oligomerization domain containing 2
6906	SERPINA7	-1.69	1.77E-03	serpin peptidase inhibitor, clade A (alpha-1 antiproteinase, antitrypsin), member 7
64902	AGXT2	-1.69	1.93E-02	alanine--glyoxylate aminotransferase 2
6362	CCL18	-1.69	7.01E-04	chemokine (C-C motif) ligand 18 (pulmonary and activation-regulated)
56521	DNAJC12	-1.69	2.28E-03	DnaJ (Hsp40) homolog, subfamily C, member 12
100423062	IGLL5	-1.69	4.02E-02	immunoglobulin lambda-like polypeptide 5
7274	TTPA	-1.68	1.32E-02	tocopherol (alpha) transfer protein
29760	BLNK	-1.68	2.88E-03	B-cell linker
6335	SCN9A	-1.68	2.84E-03	sodium channel, voltage-gated, type IX, alpha subunit
126410	CYP4F22	-1.68	2.94E-03	cytochrome P450, family 4, subfamily F, polypeptide 22
126	ADH1C	-1.67	3.50E-02	alcohol dehydrogenase 1C (class I), gamma polypeptide
10351	ABCA8	-1.67	1.30E-02	ATP-binding cassette, sub-family A (ABC1), member 8
3003	GZMK	-1.66	6.15E-03	granzyme K (granzyme 3 /// tryptase II)
9971	NR1H4	-1.66	8.69E-05	nuclear receptor subfamily 1, group H, member 4
5629	PROX1	-1.66	3.84E-05	prospero homeobox 1
388403	YPEL2	-1.65	2.42E-04	yippee-like 2 (Drosophila)
84072	HORMAD1	-1.65	4.16E-02	HORMA domain containing 1
347051	SLC10A5	-1.65	8.97E-03	solute carrier family 10, member 5
6318	SERPINB4	-1.63	2.98E-02	serpin peptidase inhibitor, clade B (ovalbumin), member 4
5858	PZP	-1.63	2.91E-03	pregnancy-zone protein
4982	TNFRSF11B	-1.63	1.41E-03	tumor necrosis factor receptor superfamily, member 11b
3429	IFI27	-1.62	1.42E-03	interferon, alpha-inducible protein 27
2621	GAS6	-1.62	1.94E-03	growth arrest-specific 6
7447	VSNL1	-1.61	1.36E-03	visinin-like 1
834	CASP1	-1.61	2.46E-02	caspase 1, apoptosis-related cysteine peptidase
6366	CCL21	-1.60	1.76E-02	chemokine (C-C motif) ligand 21
6775	STAT4	-1.60	2.49E-03	signal transducer and activator of transcription 4
84171	LOXL4	-1.60	3.32E-02	lysyl oxidase-like 4
2212	FCGR2A	-1.60	3.96E-03	Fc fragment of IgG, low affinity IIa, receptor (CD32)
10158	PDZK1IP1	-1.60	2.84E-02	PDZK1 interacting protein 1
4316	MMP7	-1.60	2.24E-02	matrix metallopeptidase 7 (matrilysin, uterine)
7097	TLR2	-1.59	2.26E-03	toll-like receptor 2
8736	MYOM1	-1.59	1.24E-02	myomesin 1
401474	SAMD12	-1.59	2.02E-02	sterile alpha motif domain containing 12
64135	IFIH1	-1.59	2.98E-03	interferon induced with helicase C domain 1
958	CD40	-1.59	1.20E-02	CD40 molecule, TNF receptor superfamily member 5
27074	LAMP3	-1.58	1.86E-04	lysosomal-associated membrane protein 3
10261	IGSF6	-1.58	2.81E-03	immunoglobulin superfamily, member 6
6890	TAP1	-1.58	3.37E-03	transporter 1, ATP-binding cassette, sub-family B (MDR/TAP)
1080	CFTR	-1.58	7.70E-04	cystic fibrosis transmembrane conductance regulator (ATP-binding cassette sub-family C, member 7)
117247	SLC16A10	-1.57	1.05E-04	solute carrier family 16 (aromatic amino acid transporter), member 10
10906	TRAFD1	-1.57	8.33E-03	TRAF-type zinc finger domain containing 1
6716	SRD5A2	-1.57	2.74E-04	steroid-5-alpha-reductase, alpha polypeptide 2 (3-oxo-5 alpha-steroid delta 4-dehydrogenase alpha 2)
400986	ANKRD36C	-1.57	4.91E-04	ankyrin repeat domain 36C
6696	SPP1	-1.57	1.41E-03	secreted phosphoprotein 1
719	C3AR1	-1.57	4.19E-03	complement component 3a receptor 1
10903	MTMR11	-1.56	2.31E-03	myotubularin related protein 11
4739	NEDD9	-1.56	1.28E-03	neural precursor cell expressed, developmentally down-regulated 9
30835	CD209	-1.56	5.51E-03	CD209 molecule
2267	FGL1	-1.56	2.06E-05	fibrinogen-like 1
3157	HMGCS1	-1.56	2.35E-05	3-hydroxy-3-methylglutaryl-CoA synthase 1 (soluble)
389197	C4orf50	-1.56	6.25E-03	chromosome 4 open reading frame 50
114836	SLAMF6	-1.56	3.72E-02	SLAM family member 6
3175	ONECUT1	-1.55	1.98E-03	one cut homeobox 1
2690	GHR	-1.55	3.72E-03	growth hormone receptor
3659	IRF1	-1.55	6.31E-03	interferon regulatory factor 1
7078	TIMP3	-1.55	9.19E-04	TIMP metallopeptidase inhibitor 3
80310	PDGFD	-1.55	9.79E-03	platelet derived growth factor D
5359	PLSCR1	-1.55	1.73E-04	phospholipid scramblase 1

4837	NNMT	-1.55	2.35E-03	nicotinamide N-methyltransferase
55013	CCDC109B	-1.55	1.04E-04	coiled-coil domain containing 109B
55076	TMEM45A	-1.55	1.12E-02	transmembrane protein 45A
3383	ICAM1	-1.55	2.24E-03	intercellular adhesion molecule 1
28755	TRAC	-1.55	1.75E-03	T cell receptor alpha constant
9627	SNCAIP	-1.54	8.39E-03	synuclein, alpha interacting protein
6772	STAT1	-1.54	1.28E-03	signal transducer and activator of transcription 1, 91kDa
994	CDC25B	-1.54	7.05E-04	cell division cycle 25B
3001	GZMA	-1.54	1.99E-02	granzyme A (granzyme 1, cytotoxic T-lymphocyte-associated serine esterase 3)
221468	TMEM217	-1.54	6.07E-03	transmembrane protein 217
117283	IP6K3	-1.54	1.18E-02	inositol hexakisphosphate kinase 3
597	BCL2A1	-1.53	8.77E-03	BCL2-related protein A1
10568	SLC34A2	-1.53	4.17E-03	solute carrier family 34 (type II sodium/phosphate contransporter), member 2
57393	TMEM27	-1.53	1.51E-02	transmembrane protein 27
10866	HCP5	-1.53	3.84E-03	HLA complex P5 (non-protein coding)
5721	PSME2	-1.53	1.29E-02	proteasome (prosome, macropain) activator subunit 2 (PA28 beta)
8564	KMO	-1.53	2.47E-03	kynurenine 3-monooxygenase (kynurenine 3-hydroxylase)
7453	WARS	-1.52	6.26E-03	tryptophanyl-tRNA synthetase
5341	PLEK	-1.52	3.76E-03	pleckstrin
55304	SPTLC3	-1.52	1.76E-03	serine palmitoyltransferase, long chain base subunit 3
2	A2M	-1.52	2.84E-02	alpha-2-macroglobulin
54625	PARP14	-1.52	6.41E-03	poly (ADP-ribose) polymerase family, member 14
338809	C12orf74	-1.51	1.54E-02	chromosome 12 open reading frame 74
9563	H6PD	-1.50	1.86E-02	hexose-6-phosphate dehydrogenase (glucose 1-dehydrogenase)
727	C5	-1.50	1.29E-02	complement component 5
9332	CD163	-1.50	1.63E-02	CD163 molecule
5696	PSMB8	-1.50	1.10E-03	proteasome (prosome, macropain) subunit, beta type, 8
80323	CCDC68	-1.50	2.66E-02	coiled-coil domain containing 68
6351	CCL4	-1.50	3.85E-02	chemokine (C-C motif) ligand 4
145864	HAPLN3	-1.49	4.00E-02	hyaluronan and proteoglycan link protein 3
54757	FAM20A	-1.49	1.00E-02	family with sequence similarity 20, member A
3075	CFH	-1.49	1.61E-04	complement factor H
59269	HIVEP3	-1.49	2.14E-03	human immunodeficiency virus type I enhancer binding protein 3
9830	TRIM14	-1.49	1.04E-04	tripartite motif containing 14
84174	SLA2	-1.49	6.31E-03	Src-like-adaptor 2
338821	SLCO1B7	-1.49	3.54E-02	solute carrier organic anion transporter family, member 1B7 (non-functional)
39	ACAT2	-1.49	9.40E-05	acetyl-CoA acetyltransferase 2
150864	FAM117B	-1.48	1.47E-03	family with sequence similarity 117, member B
1824	DSC2	-1.48	1.79E-04	desmocollin 2
64116	SLC39A8	-1.48	2.14E-03	solute carrier family 39 (zinc transporter), member 8
81788	NUAK2	-1.48	1.72E-02	NUAK family, SNF1-like kinase, 2
4261	CIITA	-1.47	2.11E-02	class II, major histocompatibility complex, transactivator
3128	HLA-DRB6	-1.47	3.96E-02	major histocompatibility complex, class II, DR beta 6 (pseudogene)
54463	FAM134B	-1.47	1.94E-03	family with sequence similarity 134, member B
50604	IL20	-1.47	4.13E-02	interleukin 20
116071	BATF2	-1.47	4.24E-02	basic leucine zipper transcription factor, ATF-like 2
8854	ALDH1A2	-1.47	2.03E-02	aldehyde dehydrogenase 1 family, member A2
6446	SGK1	-1.47	2.21E-03	serum/glucocorticoid regulated kinase 1
10384	BTN3A3	-1.47	6.77E-03	butyrophilin, subfamily 3, member A3
127733	UBXN10	-1.47	1.09E-02	UBX domain protein 10
94241	TP53INP1	-1.47	1.10E-03	tumor protein p53 inducible nuclear protein 1
5699	PSMB10	-1.46	2.00E-03	proteasome (prosome, macropain) subunit, beta type, 10
25939	SAMHD1	-1.46	1.19E-02	SAM domain and HD domain 1
843	CASP10	-1.46	1.96E-02	caspase 10, apoptosis-related cysteine peptidase
129607	CMPK2	-1.46	2.91E-02	cytidine monophosphate (UMP-CMP) kinase 2, mitochondrial
10993	SDS	-1.46	2.10E-03	serine dehydratase
158584	FAAH2	-1.46	8.28E-03	fatty acid amide hydrolase 2
113263	GLCCI1	-1.46	1.47E-03	glucocorticoid induced transcript 1
1723	DHODH	-1.46	4.59E-02	dihydroorotate dehydrogenase (quinone)
1806	DPYD	-1.46	2.10E-03	dihydropyrimidine dehydrogenase
4494	MT1F	-1.46	1.86E-04	metallothionein 1F
158219	TTC39B	-1.46	6.77E-03	tetratricopeptide repeat domain 39B
55603	FAM46A	-1.46	8.97E-03	family with sequence similarity 46, member A
81794	ADAMTS10	-1.46	7.03E-04	ADAM metallopeptidase with thrombospondin type 1 motif, 10
28638	TRBC2	-1.46	2.60E-02	T cell receptor beta constant 2
128439	SNHG11	-1.46	1.72E-02	small nucleolar RNA host gene 11 (non-protein coding)
51313	FAM198B	-1.46	4.69E-03	family with sequence similarity 198, member B
8600	TNFSF11	-1.46	2.18E-03	tumor necrosis factor (ligand) superfamily, member 11
10154	PLXNC1	-1.45	1.31E-02	plexin C1
168537	GIMAP7	-1.45	4.60E-03	GTPase, IMAP family member 7
57674	RNF213	-1.45	2.89E-03	ring finger protein 213
10225	CD96	-1.45	5.43E-03	CD96 molecule
55075	UACA	-1.45	3.09E-03	uveal autoantigen with coiled-coil domains and ankyrin repeats
2272	FHIT	-1.45	4.50E-02	fragile histidine triad
84419	C15orf48	-1.45	2.99E-02	chromosome 15 open reading frame 48
10379	IRF9	-1.45	7.70E-04	interferon regulatory factor 9
3431	SP110	-1.45	2.58E-03	SP110 nuclear body protein
100133315	LOC100133315	-1.44	2.60E-02	transient receptor potential cation channel, subfamily C, member 2-like
406	ARNTL	-1.44	5.20E-03	aryl hydrocarbon receptor nuclear translocator-like
3005	H1F0	-1.44	7.10E-04	H1 histone family, member 0
337972	KRTAP19-5	-1.44	4.67E-02	keratin associated protein 19-5
3728	JUP	-1.44	2.78E-04	junction plakoglobin
2160	F11	-1.44	6.75E-04	coagulation factor XI
23213	SULF1	-1.44	1.39E-02	sulfatase 1
953	ENTPD1	-1.44	4.64E-03	ectonucleoside triphosphate diphosphohydrolase 1
6385	SDC4	-1.44	1.78E-03	syndecan 4
1520	CTSS	-1.44	2.65E-03	cathepsin S
259307	IL4I1	-1.44	8.20E-03	interleukin 4 induced 1

3929	LBP	-1.44	1.77E-03	lipopolysaccharide binding protein
440836	ODF3B	-1.44	3.11E-02	outer dense fiber of sperm tails 3B
6590	SLPI	-1.43	2.71E-03	secretory leukocyte peptidase inhibitor
6499	SKIV2L	-1.43	1.12E-02	superkiller viralicidic activity 2-like (S. cerevisiae)
199	AIF1	-1.43	1.64E-02	allograft inflammatory factor 1
4008	LMO7	-1.42	1.97E-03	LIM domain 7
23460	ABCA6	-1.42	1.08E-02	ATP-binding cassette, sub-family A (ABC1), member 6
4318	MMP9	-1.42	6.31E-03	matrix metallopeptidase 9 (gelatinase B, 92kDa gelatinase, 92kDa type IV collagenase)
8832	CD84	-1.42	5.45E-03	CD84 molecule
5031	P2RY6	-1.42	3.42E-02	pyrimidinergic receptor P2Y, G-protein coupled, 6
79132	DHX58	-1.42	3.43E-02	DEXH (Asp-Glu-X-His) box polypeptide 58
8013	NR4A3	-1.42	4.19E-03	nuclear receptor subfamily 4, group A, member 3
23012	STK38L	-1.42	4.42E-05	serine/threonine kinase 38 like
2766	GMPR	-1.42	4.93E-02	guanosine monophosphate reductase
6892	TAPBP	-1.42	1.59E-04	TAP binding protein (tapasin)
25816	TNFAIP8	-1.42	8.39E-03	tumor necrosis factor, alpha-induced protein 8
91351	DDX60L	-1.42	2.46E-02	DEAD (Asp-Glu-Ala-Asp) box polypeptide 60-like
1643	DDB2	-1.42	2.30E-03	damage-specific DNA binding protein 2, 48kDa
23780	APOL2	-1.42	4.60E-03	apolipoprotein L, 2
7412	VCAM1	-1.41	4.45E-02	vascular cell adhesion molecule 1
3113	HLA-DPA1	-1.41	5.99E-03	major histocompatibility complex, class II, DP alpha 1
132321	C4orf33	-1.41	2.50E-02	chromosome 4 open reading frame 33
51348	KLRF1	-1.41	1.38E-02	killer cell lectin-like receptor subfamily F, member 1
58476	TP53INP2	-1.41	6.07E-03	tumor protein p53 inducible nuclear protein 2
976	CD97	-1.41	4.10E-02	CD97 molecule
3176	HNMT	-1.41	6.89E-04	histamine N-methyltransferase
83875	BCO2	-1.41	2.47E-02	beta-carotene oxygenase 2
3140	MR1	-1.41	4.81E-03	major histocompatibility complex, class I-related
1236	CCR7	-1.41	4.59E-02	chemokine (C-C motif) receptor 7
166336	PRICKLE2	-1.40	3.02E-02	prickle homolog 2 (Drosophila)
56892	C8orf4	-1.40	1.14E-03	chromosome 8 open reading frame 4
83593	RASSF5	-1.40	2.72E-02	Ras association (RalGDS/AF-6) domain family member 5
79668	PARP8	-1.40	4.01E-03	poly (ADP-ribose) polymerase family, member 8
10410	IFITM3	-1.39	4.62E-04	interferon induced transmembrane protein 3
9235	IL32	-1.39	1.01E-03	interleukin 32
55064	SPATA6L	-1.39	1.86E-02	spermatogenesis associated 6-like
6480	ST6GAL1	-1.39	6.72E-03	ST6 beta-galactosamide alpha-2,6-sialyltranferase 1
9746	CLSTN3	-1.39	1.04E-04	calsyntenin 3
4343	MOV10	-1.39	1.30E-03	Mov10, Moloney leukemia virus 10, homolog (mouse)
255231	MCOLN2	-1.39	3.34E-02	mucolipin 2
6695	SPOCK1	-1.39	8.47E-03	sparc/osteonectin, cwcv and kazal-like domains proteoglycan (testican) 1
6718	AKR1D1	-1.39	2.41E-03	aldo-keto reductase family 1, member D1
10100	TSPAN2	-1.38	3.56E-02	tetraspanin 2
30817	EMR2	-1.38	9.46E-03	egf-like module containing, mucin-like, hormone receptor-like 2
3717	JAK2	-1.38	2.08E-02	Janus kinase 2
79776	ZFHX4	-1.38	6.71E-03	zinc finger homeobox 4
348645	C22orf34	-1.38	4.61E-02	chromosome 22 open reading frame 34
91937	TIMD4	-1.38	1.30E-02	T-cell immunoglobulin and mucin domain containing 4
1033	CDKN3	-1.38	4.83E-02	cyclin-dependent kinase inhibitor 3
10863	ADAM28	-1.38	2.08E-02	ADAM metallopeptidase domain 28
93663	ARHGAP18	-1.38	4.55E-03	Rho GTPase activating protein 18
1073	CFL2	-1.38	7.21E-04	cofilin 2 (muscle)
64218	SEMA4A	-1.38	1.89E-03	sema domain, immunoglobulin domain (Ig), transmembrane domain (TM) and short cytoplasmic domain, (semaphorin) 4A
26031	OSBPL3	-1.38	1.69E-02	oxysterol binding protein-like 3
148213	ZNF681	-1.38	2.66E-02	zinc finger protein 681
26191	PTPN22	-1.38	4.51E-02	protein tyrosine phosphatase, non-receptor type 22 (lymphoid)
140564	APOBEC3D	-1.38	3.00E-02	apolipoprotein B mRNA editing enzyme, catalytic polypeptide-like 3D
401494	PTPLAD2	-1.37	3.49E-02	protein tyrosine phosphatase-like A domain containing 2
6653	SORL1	-1.37	1.38E-02	sortilin-related receptor, L(DLR class) A repeats containing
7903	ST8SIA4	-1.37	1.62E-02	ST8 alpha-N-acetyl-neuraminide alpha-2,8-sialyltransferase 4
55824	PAG1	-1.37	1.82E-02	phosphoprotein associated with glycosphingolipid microdomains 1
9246	UBE2L6	-1.37	1.62E-02	ubiquitin-conjugating enzyme E2L 6
6347	CCL2	-1.37	1.39E-02	chemokine (C-C motif) ligand 2
145957	NRG4	-1.37	2.78E-02	neuregulin 4
6891	TAP2	-1.37	4.26E-02	transporter 2, ATP-binding cassette, sub-family B (MDR/TAP)
3026	HABP2	-1.37	1.59E-03	hyaluronan binding protein 2
81704	DOCK8	-1.37	8.38E-03	dedicator of cytokinesis 8
9175	MAP3K13	-1.37	4.25E-02	mitogen-activated protein kinase kinase kinase 13
10457	GPNMB	-1.37	3.71E-02	glycoprotein (transmembrane) nmb
79370	BCL2L14	-1.37	2.73E-02	BCL2-like 14 (apoptosis facilitator)
4084	MXD1	-1.37	9.72E-04	MAX dimerization protein 1
3070	HELLS	-1.37	4.87E-02	helicase, lymphoid-specific
5880	RAC2	-1.37	9.40E-03	ras-related C3 botulinum toxin substrate 2 (rho family, small GTP binding protein Rac2)
5027	P2RX7	-1.37	8.81E-03	purinergic receptor P2X, ligand-gated ion channel, 7
857	CAV1	-1.36	1.64E-02	caveolin 1, caveolae protein, 22kDa
10203	CALCRL	-1.36	1.77E-02	calcitonin receptor-like
2207	FCER1G	-1.36	9.73E-03	Fc fragment of IgE, high affinity I, receptor for /// gamma polypeptide
8502	PKP4	-1.36	5.46E-04	plakophilin 4
8836	GGH	-1.36	2.47E-02	gamma-glutamyl hydrolase (conjugase, folylpolygammaglutamyl hydrolase)
90527	DUOXA1	-1.36	7.43E-03	dual oxidase maturation factor 1
8673	VAMP8	-1.36	7.98E-05	vesicle-associated membrane protein 8
3122	HLA-DRA	-1.36	3.73E-03	major histocompatibility complex, class II, DR alpha
123803	NTAN1	-1.36	4.53E-03	N-terminal asparagine amidase
94015	TTYH2	-1.36	2.56E-03	tweety family member 2
3604	TNFRSF9	-1.36	1.83E-02	tumor necrosis factor receptor superfamily, member 9
10288	LILRB2	-1.36	4.75E-02	leukocyte immunoglobulin-like receptor, subfamily B (with TM and ITIM domains), member 2
481	ATP1B1	-1.36	2.68E-03	ATPase, Na+/K+ transporting, beta 1 polypeptide
10581	IFITM2	-1.36	1.13E-02	interferon induced transmembrane protein 2

23648	SSBP3	-1.36	1.11E-02	single stranded DNA binding protein 3
64092	SAMSN1	-1.35	5.45E-03	SAM domain, SH3 domain and nuclear localization signals 1
342132	ZNF774	-1.35	4.35E-02	zinc finger protein 774
57169	ZNFX1	-1.35	8.82E-03	zinc finger, NFX1-type containing 1
4588	MUC6	-1.35	4.84E-02	mucin 6, oligomeric mucus/gel-forming
10133	OPTN	-1.35	1.06E-03	optineurin
55509	BATF3	-1.35	7.56E-03	basic leucine zipper transcription factor, ATF-like 3
23768	FLRT2	-1.35	5.43E-03	fibronectin leucine rich transmembrane protein 2
961	CD47	-1.35	5.96E-03	CD47 molecule
1767	DNAH5	-1.35	3.89E-02	dynein, axonemal, heavy chain 5
84166	NLRC5	-1.35	3.43E-02	NLR family, CARD domain containing 5
241	ALOX5AP	-1.35	8.51E-03	arachidonate 5-lipoxygenase-activating protein
2627	GATA6	-1.35	1.44E-02	GATA binding protein 6
50515	CHST11	-1.34	7.48E-04	carbohydrate (chondroitin 4) sulfotransferase 11
1519	CTSO	-1.34	1.64E-02	cathepsin O
669	BPGM	-1.34	1.06E-03	2,3-bisphosphoglycerate mutase
7111	TMOD1	-1.34	2.87E-02	tropomodulin 1
6367	CCL22	-1.34	1.96E-02	chemokine (C-C motif) ligand 22
51704	GPRC5B	-1.34	1.34E-03	G protein-coupled receptor, class C, group 5, member B
38	ACAT1	-1.34	1.27E-03	acetyl-CoA acetyltransferase 1
725	C4BPB	-1.34	3.36E-02	complement component 4 binding protein, beta
972	CD74	-1.34	7.61E-03	CD74 molecule, major histocompatibility complex, class II invariant chain
89870	TRIM15	-1.34	3.61E-02	tripartite motif containing 15
1004	CDH6	-1.34	9.60E-03	cadherin 6, type 2, K-cadherin (fetal kidney)
25903	OLFML2B	-1.34	4.67E-02	olfactomedin-like 2B
9223	MAGI1	-1.34	4.24E-03	membrane associated guanylate kinase, WW and PDZ domain containing 1
164656	TMPRSS6	-1.33	1.44E-03	transmembrane protease, serine 6
85441	HELZ2	-1.33	2.85E-02	helicase with zinc finger 2, transcriptional coactivator
7456	WIPF1	-1.33	2.23E-02	WAS/WASL interacting protein family, member 1
55619	DOCK10	-1.33	1.64E-02	dedicator of cytokinesis 10
57530	CGN	-1.33	6.26E-03	cingulin
3108	HLA-DMA	-1.33	1.63E-02	major histocompatibility complex, class II, DM alpha
57619	SHROOM3	-1.33	2.47E-02	shroom family member 3
1880	GPR183	-1.33	1.17E-02	G protein-coupled receptor 183
1890	TYMP	-1.33	6.65E-03	thymidine phosphorylase
7743	ZNF189	-1.33	4.89E-03	zinc finger protein 189
4300	MLLT3	-1.33	3.98E-02	myeloid/lymphoid or mixed-lineage leukemia (trithorax homolog, Drosophila) /// translocated to, 3
728411	GUSBP1	-1.33	6.77E-03	glucuronidase, beta pseudogene 1
57655	GRAMD1A	-1.32	2.19E-02	GRAM domain containing 1A
151636	DTX3L	-1.32	6.37E-03	deltex 3 like, E3 ubiquitin ligase
3937	LCP2	-1.32	1.93E-02	lymphocyte cytosolic protein 2 (SH2 domain containing leukocyte protein of 76kDa)
11199	ANXA10	-1.32	2.97E-02	annexin A10
5734	PTGER4	-1.32	1.75E-02	prostaglandin E receptor 4 (subtype EP4)
920	CD4	-1.32	2.47E-02	CD4 molecule
64393	ZMAT3	-1.32	1.59E-03	zinc finger, matrin-type 3
80765	STARD5	-1.32	4.37E-02	StAR-related lipid transfer (START) domain containing 5
23338	JADE2	-1.32	3.53E-02	jade family PHD finger 2
717	C2	-1.32	5.70E-04	complement component 2
694	BTG1	-1.32	1.52E-04	B-cell translocation gene 1, anti-proliferative
5552	SRGN	-1.32	1.46E-03	serglycin
64167	ERAP2	-1.32	3.83E-02	endoplasmic reticulum aminopeptidase 2
7104	TM4SF4	-1.32	3.42E-02	transmembrane 4 L six family member 4
8869	ST3GAL5	-1.32	4.11E-02	ST3 beta-galactoside alpha-2,3-sialyltransferase 5
18	ABAT	-1.32	1.29E-02	4-aminobutyrate aminotransferase
11067	C10orf10	-1.31	1.35E-03	chromosome 10 open reading frame 10
359845	FAM101B	-1.31	6.38E-03	family with sequence similarity 101, member B
1030	CDKN2B	-1.31	8.76E-03	cyclin-dependent kinase inhibitor 2B (p15, inhibits CDK4)
5294	PIK3CG	-1.31	3.94E-02	phosphatidylinositol-4,5-bisphosphate 3-kinase, catalytic subunit gamma
133308	SLC9B2	-1.31	2.05E-03	solute carrier family 9, subfamily B (NHA2, cation proton antiporter 2), member 2
51312	SLC25A37	-1.31	9.47E-03	solute carrier family 25 (mitochondrial iron transporter), member 37
5918	RARRES1	-1.31	2.04E-02	retinoic acid receptor responder (tazarotene induced) 1
126917	IFFO2	-1.31	2.58E-02	intermediate filament family orphan 2
135112	NCOA7	-1.31	7.35E-03	nuclear receptor coactivator 7
9873	FCHSD2	-1.31	5.67E-03	FCH and double SH3 domains 2
64856	VWA1	-1.31	3.68E-02	von Willebrand factor A domain containing 1
5788	PTPRC	-1.31	4.67E-02	protein tyrosine phosphatase, receptor type, C
1356	CP	-1.31	5.03E-03	ceruloplasmin (ferroxidase)
4524	MTHFR	-1.31	1.86E-02	methylenetetrahydrofolate reductase (NAD(P)H)
6609	SMPD1	-1.30	1.40E-02	sphingomyelin phosphodiesterase 1, acid lysosomal
79895	ATP8B4	-1.30	5.00E-02	ATPase, class I, type 8B, member 4
2595	GANC	-1.30	2.92E-02	glucosidase, alpha /// neutral C
51279	C1RL	-1.30	5.89E-03	complement component 1, r subcomponent-like
732	C8B	-1.30	4.99E-03	complement component 8, beta polypeptide
1500	CTNND1	-1.30	5.77E-03	catenin (cadherin-associated protein), delta 1
6567	SLC16A2	-1.30	1.86E-02	solute carrier family 16, member 2 (thyroid hormone transporter)
57185	NIPAL3	-1.30	2.98E-02	NIPA-like domain containing 3
2040	STOM	-1.30	3.61E-03	stomatin
6387	CXCL12	-1.30	1.30E-03	chemokine (C-X-C motif) ligand 12
200316	APOBEC3F	-1.30	1.49E-02	apolipoprotein B mRNA editing enzyme, catalytic polypeptide-like 3F
3115	HLA-DPB1	-1.30	3.01E-02	major histocompatibility complex, class II, DP beta 1
118788	PIK3AP1	-1.29	2.62E-02	phosphoinositide-3-kinase adaptor protein 1
23094	SIPA1L3	-1.29	3.83E-03	signal-induced proliferation-associated 1 like 3
118429	ANTXR2	-1.29	6.39E-04	anthrax toxin receptor 2
4430	MYO1B	-1.29	1.60E-03	myosin IB
63901	FAM111A	-1.29	2.75E-02	family with sequence similarity 111, member A
5796	PTPRK	-1.29	1.30E-03	protein tyrosine phosphatase, receptor type, K
5519	PPP2R1B	-1.29	1.27E-03	protein phosphatase 2, regulatory subunit A, beta
55902	ACSS2	-1.29	1.23E-02	acyl-CoA synthetase short-chain family member 2

53	ACP2	-1.29	5.23E-03	acid phosphatase 2, lysosomal
9881	TRANK1	-1.29	2.08E-02	tetratricopeptide repeat and ankyrin repeat containing 1
27106	ARRDC2	-1.29	1.00E-02	arrestin domain containing 2
2266	FGG	-1.28	6.80E-03	fibrinogen gamma chain
10039	PARP3	-1.28	1.30E-02	poly (ADP-ribose) polymerase family, member 3
23424	TDRD7	-1.28	3.10E-02	tudor domain containing 7
9674	KIAA0040	-1.28	1.07E-02	KIAA0040
10026	PIGK	-1.28	3.82E-02	phosphatidylinositol glycan anchor biosynthesis, class K
11221	DUSP10	-1.28	8.93E-03	dual specificity phosphatase 10
822	CAPG	-1.28	2.50E-02	capping protein (actin filament), gelsolin-like
54557	SGTB	-1.28	4.05E-02	small glutamine-rich tetratricopeptide repeat (TPR)-containing, beta
285830	HLA-F-AS1	-1.28	4.86E-02	HLA-F antisense RNA 1
2028	ENPEP	-1.28	3.11E-02	glutamyl aminopeptidase (aminopeptidase A)
23235	SIK2	-1.28	3.77E-02	salt-inducible kinase 2
1959	EGR2	-1.28	1.61E-02	early growth response 2
11151	CORO1A	-1.28	1.86E-02	coronin, actin binding protein, 1A
55620	STAP2	-1.28	2.36E-02	signal transducing adaptor family member 2
5045	FURIN	-1.28	6.20E-03	furin (paired basic amino acid cleaving enzyme)
10437	IFI30	-1.28	4.21E-02	interferon, gamma-inducible protein 30
726	CAPN5	-1.28	2.02E-02	calpain 5
3134	HLA-F	-1.28	3.22E-02	major histocompatibility complex, class I, F
9032	TM4SF5	-1.28	2.96E-02	transmembrane 4 L six family member 5
23705	CADM1	-1.28	4.25E-03	cell adhesion molecule 1
54464	XRN1	-1.28	1.55E-02	5'-3' exoribonuclease 1
5720	PSME1	-1.27	6.25E-03	proteasome (prosome, macropain) activator subunit 1 (PA28 alpha)
3096	HIVEP1	-1.27	1.18E-03	human immunodeficiency virus type I enhancer binding protein 1
5345	SERPINF2	-1.27	2.14E-02	serpin peptidase inhibitor, clade F (alpha-2 antiplasmin, pigment epithelium derived factor), member 2
1512	CTSH	-1.27	2.57E-03	cathepsin H
1803	DPP4	-1.27	2.41E-02	dipeptidyl-peptidase 4
90139	TSPAN18	-1.27	3.62E-02	tetraspanin 18
6615	SNAI1	-1.27	4.10E-02	snail family zinc finger 1
51542	VPS54	-1.27	3.68E-02	vacuolar protein sorting 54 homolog (S. cerevisiae)
5058	PAK1	-1.27	1.28E-02	p21 protein (Cdc42/Rac)-activated kinase 1
11161	C14orf1	-1.27	1.39E-02	chromosome 14 open reading frame 1
5610	EIF2AK2	-1.27	1.93E-02	eukaryotic translation initiation factor 2-alpha kinase 2
115825	WDFY2	-1.27	3.42E-03	WD repeat and FYVE domain containing 2
5337	PLD1	-1.27	4.48E-02	phospholipase D1, phosphatidylcholine-specific
8542	APOL1	-1.27	3.17E-02	apolipoprotein L, 1
9926	LPGAT1	-1.27	2.96E-02	lysophosphatidylglycerol acyltransferase 1
11278	KLF12	-1.27	3.84E-02	Kruppel-like factor 12
374378	GALNT18	-1.27	3.42E-02	UDP-N-acetyl-alpha-D-galactosamine:polypeptide N-acetylgalactosaminyltransferase 18
23654	PLXNB2	-1.27	1.86E-04	plexin B2
56829	ZC3HAV1	-1.27	1.77E-03	zinc finger CCCH-type, antiviral 1
6240	RRM1	-1.27	4.26E-02	ribonucleotide reductase M1
5641	LGMN	-1.26	7.37E-03	legumain
8148	TAF15	-1.26	7.03E-04	TAF15 RNA polymerase II, TATA box binding protein (TBP)-associated factor, 68kDa
84159	ARID5B	-1.26	2.67E-03	AT rich interactive domain 5B (MRF1-like)
3587	IL10RA	-1.26	2.51E-02	interleukin 10 receptor, alpha
221895	JAZF1	-1.26	4.96E-02	JAZF zinc finger 1
124540	MSI2	-1.26	1.18E-02	musashi RNA-binding protein 2
3683	ITGAL	-1.26	4.54E-02	integrin, alpha L (antigen CD11A (p180), lymphocyte function-associated antigen 1 /// alpha polypeptide)
2934	GSN	-1.26	1.71E-02	gelsolin
8530	CST7	-1.26	3.33E-02	cystatin F (leukocystatin)
6768	ST14	-1.26	2.41E-02	suppression of tumorigenicity 14 (colon carcinoma)
285440	CYP4V2	-1.26	1.17E-03	cytochrome P450, family 4, subfamily V, polypeptide 2
64780	MICAL1	-1.26	4.29E-02	microtubule associated monooxygenase, calponin and LIM domain containing 1
629	CFB	-1.26	6.18E-04	complement factor B
3732	CD82	-1.26	1.53E-02	CD82 molecule
9945	GFPT2	-1.26	4.62E-02	glutamine-fructose-6-phosphate transaminase 2
64761	PARP12	-1.26	6.07E-03	poly (ADP-ribose) polymerase family, member 12
6307	MSMO1	-1.26	3.17E-02	methylsterol monooxygenase 1
85461	TANC1	-1.26	8.85E-03	tetratricopeptide repeat, ankyrin repeat and coiled-coil containing 1
81553	FAM49A	-1.26	4.31E-02	family with sequence similarity 49, member A
407018	MIR27A	-1.26	3.53E-02	microRNA 27a
9414	TJP2	-1.26	1.15E-02	tight junction protein 2
3684	ITGAM	-1.26	1.83E-02	integrin, alpha M (complement component 3 receptor 3 subunit)
10018	BCL2L11	-1.26	1.86E-02	BCL2-like 11 (apoptosis facilitator)
5654	HTRA1	-1.26	1.83E-02	HtrA serine peptidase 1
2995	GYPC	-1.26	4.35E-03	glycophorin C (Gerbich blood group)
718	C3	-1.26	1.59E-03	complement component 3
51474	LIMA1	-1.26	1.00E-02	LIM domain and actin binding 1
51667	NUB1	-1.25	8.02E-03	negative regulator of ubiquitin-like proteins 1
2037	EPB41L2	-1.25	4.73E-02	erythrocyte membrane protein band 4.1-like 2
3507	IGHM	-1.25	3.73E-02	immunoglobulin heavy constant mu
10982	MAPRE2	-1.25	2.70E-02	microtubule-associated protein, RP/EB family, member 2
716	C1S	-1.25	3.37E-03	complement component 1, s subcomponent
84885	ZDHHC12	-1.25	2.65E-02	zinc finger, DHHC-type containing 12
10157	AASS	-1.25	3.95E-02	aminoadipate-semialdehyde synthase
10420	TESK2	-1.25	3.36E-02	testis-specific kinase 2
23136	EPB41L3	-1.25	2.40E-02	erythrocyte membrane protein band 4.1-like 3
4853	NOTCH2	-1.25	1.08E-02	notch 2
80830	APOL6	-1.25	3.44E-02	apolipoprotein L, 6
51768	TM7SF3	-1.25	8.33E-03	transmembrane 7 superfamily member 3
5993	RFX5	-1.25	4.03E-02	regulatory factor X, 5 (influences HLA class II expression)
3426	CFI	-1.25	2.51E-02	complement factor I
868	CBLB	-1.25	2.22E-03	Cbl proto-oncogene B, E3 ubiquitin protein ligase
55281	TMEM140	-1.25	2.80E-02	transmembrane protein 140
4067	LYN	-1.25	4.68E-03	v-yes-1 Yamaguchi sarcoma viral related oncogene homolog

5538	PPT1	-1.25	2.81E-02	palmitoyl-protein thioesterase 1
4818	NKG7	-1.25	4.68E-02	natural killer cell granule protein 7
7277	TUBA4A	-1.24	2.91E-02	tubulin, alpha 4a
55337	C19orf66	-1.24	1.36E-02	chromosome 19 open reading frame 66
93349	SP140L	-1.24	4.37E-02	SP140 nuclear body protein-like
3371	TNC	-1.24	2.34E-02	tenascin C
330	BIRC3	-1.24	3.31E-02	baculoviral IAP repeat containing 3
123036	TC2N	-1.24	3.98E-02	tandem C2 domains, nuclear
3601	IL15RA	-1.24	7.43E-03	interleukin 15 receptor, alpha
23253	ANKRD12	-1.24	4.68E-03	ankyrin repeat domain 12
2224	FDPS	-1.24	4.91E-02	farnesyl diphosphate synthase
134429	STARD4	-1.24	2.67E-03	StAR-related lipid transfer (START) domain containing 4
57381	RHOJ	-1.24	2.10E-02	ras homolog family member J
4891	SLC11A2	-1.24	9.74E-03	solute carrier family 11 (proton-coupled divalent metal ion transporter), member 2
4647	MYO7A	-1.24	2.91E-02	myosin VIIA
89796	NAV1	-1.24	1.03E-02	neuron navigator 1
8728	ADAM19	-1.24	1.06E-02	ADAM metallopeptidase domain 19
23157	6-sep	-1.24	1.54E-02	septin 6
12	SERPINA3	-1.24	1.80E-02	serpin peptidase inhibitor, clade A (alpha-1 antiproteinase, antitrypsin), member 3
84230	LRRC8C	-1.23	9.60E-03	leucine rich repeat containing 8 family, member C
4124	MAN2A1	-1.23	3.37E-03	mannosidase, alpha, class 2A, member 1
10046	MAMLD1	-1.23	8.96E-03	mastermind-like domain containing 1
3588	IL10RB	-1.23	1.10E-02	interleukin 10 receptor, beta
3017	HIST1H2BD	-1.23	2.26E-02	histone cluster 1, H2bd
260425	MAGI3	-1.23	1.77E-02	membrane associated guanylate kinase, WW and PDZ domain containing 3
123096	SLC25A29	-1.23	3.85E-02	solute carrier family 25 (mitochondrial carnitine/acylcarnitine carrier), member 29
23635	SSBP2	-1.23	2.26E-02	single-stranded DNA binding protein 2
22920	KIFAP3	-1.23	1.16E-02	kinesin-associated protein 3
3398	ID2	-1.23	1.82E-02	inhibitor of DNA binding 2, dominant negative helix-loop-helix protein
9766	KIAA0247	-1.23	2.65E-02	KIAA0247
214	ALCAM	-1.23	2.19E-02	activated leukocyte cell adhesion molecule
23406	COTL1	-1.23	4.73E-02	coactosin-like F-actin binding protein 1
10957	PNRC1	-1.23	6.43E-03	proline-rich nuclear receptor coactivator 1
8613	PPAP2B	-1.23	3.98E-02	phosphatidic acid phosphatase type 2B
140606	SELM	-1.23	3.77E-03	selenoprotein M
23033	DOPEY1	-1.23	3.17E-02	dopey family member 1
178	AGL	-1.23	3.00E-02	amylo-alpha-1, 6-glucosidase, 4-alpha-glucanotransferase
4704	NDUFA9	-1.23	3.61E-02	NADH dehydrogenase (ubiquinone) 1 alpha subcomplex, 9, 39kDa
27244	SESN1	-1.22	4.68E-02	sestrin 1
7263	TST	-1.22	3.46E-02	thiosulfate sulfurtransferase (rhodanese)
23293	SMG6	-1.22	3.47E-02	SMG6 nonsense mediated mRNA decay factor
8844	KSR1	-1.22	1.41E-02	kinase suppressor of ras 1
2683	B4GALT1	-1.22	4.54E-02	UDP-Gal:betaGlcNAc beta 1,4- galactosyltransferase, polypeptide 1
659	BMPR2	-1.22	1.23E-02	bone morphogenetic protein receptor, type II (serine/threonine kinase)
9021	SOCS3	-1.22	2.80E-02	suppressor of cytokine signaling 3
11031	RAB31	-1.22	1.03E-02	RAB31, member RAS oncogene family
114569	MAL2	-1.22	6.25E-03	mal, T-cell differentiation protein 2 (gene/pseudogene)
5156	PDGFRA	-1.22	3.54E-02	platelet-derived growth factor receptor, alpha polypeptide
51170	HSD17B11	-1.22	3.96E-02	hydroxysteroid (17-beta) dehydrogenase 11
29992	PILRA	-1.22	2.80E-02	paired immunoglobin-like type 2 receptor alpha
56927	GPR108	-1.22	4.31E-02	G protein-coupled receptor 108
79815	NIPAL2	-1.22	4.96E-02	NIPA-like domain containing 2
23604	DAPK2	-1.21	4.96E-02	death-associated protein kinase 2
80344	DCAF11	-1.21	1.86E-02	DDB1 and CUL4 associated factor 11
2647	BLOC1S1	-1.21	2.97E-02	biogenesis of lysosomal organelles complex-1, subunit 1
3428	IFI16	-1.21	4.83E-02	interferon, gamma-inducible protein 16
55803	ADAP2	-1.21	3.48E-02	ArfGAP with dual PH domains 2
196527	ANO6	-1.21	6.48E-03	anoctamin 6
307	ANXA4	-1.21	4.24E-02	annexin A4
285513	GPRIN3	-1.21	3.34E-02	GPRIN family member 3
9770	RASSF2	-1.21	1.06E-02	Ras association (RalGDS/AF-6) domain family member 2
647135	SRGAP2B	-1.21	9.80E-03	SLIT-ROBO Rho GTPase activating protein 2B
355	FAS	-1.21	2.11E-02	Fas cell surface death receptor
3087	HHEX	-1.21	1.57E-02	hematopoietically expressed homeobox
91452	ACBD5	-1.21	2.80E-02	acyl-CoA binding domain containing 5
55748	CNDP2	-1.21	3.73E-02	CNDP dipeptidase 2 (metallopeptidase M20 family)
6672	SP100	-1.20	1.99E-02	SP100 nuclear antigen
11329	STK38	-1.20	1.79E-02	serine/threonine kinase 38
88455	ANKRD13A	-1.20	4.01E-03	ankyrin repeat domain 13A
4478	MSN	-1.20	7.81E-03	moesin
2581	GALC	-1.20	4.37E-02	galactosylceramidase
80021	TMEM62	-1.20	4.23E-02	transmembrane protein 62
10769	PLK2	-1.20	1.32E-02	polo-like kinase 2
79056	PRRG4	-1.20	2.81E-02	proline rich Gla (G-carboxyglutamic acid) 4 (transmembrane)
900	CCNG1	-1.20	2.47E-03	cyclin G1
25937	WWTR1	-1.20	3.42E-02	WW domain containing transcription regulator 1
9124	PDLIM1	-1.19	3.67E-02	PDZ and LIM domain 1
722	C4BPA	-1.19	9.29E-03	complement component 4 binding protein, alpha
57186	RALGAPA2	-1.19	2.91E-02	Ral GTPase activating protein, alpha subunit 2 (catalytic)
7052	TGM2	-1.19	3.50E-02	transglutaminase 2
23102	TBC1D2B	-1.19	4.98E-02	TBC1 domain family, member 2B
6709	SPTAN1	-1.19	1.15E-02	spectrin, alpha, non-erythrocytic 1
1730	DIAPH2	-1.19	2.42E-02	diaphanous-related formin 2
6282	S100A11	-1.19	3.86E-02	S100 calcium binding protein A11
6934	TCF7L2	-1.19	1.14E-02	transcription factor 7-like 2 (T-cell specific, HMG-box)
23516	SLC39A14	-1.18	4.82E-02	solute carrier family 39 (zinc transporter), member 14
4646	MYO6	-1.18	1.55E-02	myosin VI
223082	ZNRF2	-1.18	3.89E-02	zinc and ring finger 2

59338	PLEKHA1	-1.18	2.64E-02	pleckstrin homology domain containing, family A (phosphoinositide binding specific) member 1
7538	ZFP36	-1.18	2.60E-02	ZFP36 ring finger protein
23646	PLD3	-1.18	2.50E-02	phospholipase D family, member 3
3065	HDAC1	-1.18	6.61E-03	histone deacetylase 1
55014	STX17	-1.18	2.47E-02	syntaxin 17
6774	STAT3	-1.18	1.00E-02	signal transducer and activator of transcription 3 (acute-phase response factor)
9500	MAGED1	-1.18	3.11E-02	melanoma antigen family D, 1
5446	PON3	-1.18	3.44E-02	paraoxonase 3
115548	FCHO2	-1.17	2.96E-02	FCH domain only 2
51088	KLHL5	-1.17	4.52E-02	kelch-like family member 5
3985	LIMK2	-1.17	2.46E-02	LIM domain kinase 2
25865	PRKD2	-1.17	2.58E-02	protein kinase D2
27436	EML4	-1.17	2.23E-02	echinoderm microtubule associated protein like 4
1021	CDK6	-1.17	4.10E-02	cyclin-dependent kinase 6
808	CALM3	-1.17	4.03E-02	calmodulin 3 (phosphorylase kinase, delta)
3699	ITIH3	-1.17	2.03E-02	inter-alpha-trypsin inhibitor heavy chain 3
8892	EIF2B2	-1.17	1.37E-02	eukaryotic translation initiation factor 2B, subunit 2 beta, 39kDa
6558	SLC12A2	-1.17	4.60E-02	solute carrier family 12 (sodium/potassium/chloride transporter), member 2
6594	SMARCA1	-1.17	3.84E-02	SWI/SNF related, matrix associated, actin dependent regulator of chromatin, subfamily a, member 1
3106	HLA-B	-1.17	3.84E-02	major histocompatibility complex, class I, B
800	CALD1	-1.17	2.52E-02	caldesmon 1
51571	FAM49B	-1.17	2.77E-02	family with sequence similarity 49, member B
731	C8A	-1.17	3.49E-02	complement component 8, alpha polypeptide
5562	PRKAA1	-1.17	2.65E-02	protein kinase, AMP-activated, alpha 1 catalytic subunit
567	B2M	-1.17	4.48E-02	beta-2-microglobulin
3611	ILK	-1.16	1.32E-02	integrin-linked kinase
23530	NNT	-1.16	2.53E-02	nicotinamide nucleotide transhydrogenase
667	DST	-1.16	2.80E-02	dystonin
30844	EHD4	-1.16	2.98E-02	EH-domain containing 4
11059	WWP1	-1.15	3.49E-02	WW domain containing E3 ubiquitin protein ligase 1
48	ACO1	-1.15	4.78E-02	aconitase 1, soluble
7317	UBA1	-1.15	2.60E-02	ubiquitin-like modifier activating enzyme 1
1282	COL4A1	-1.15	4.54E-02	collagen, type IV, alpha 1
9879	DDX46	-1.15	4.03E-02	DEAD (Asp-Glu-Ala-Asp) box polypeptide 46
4670	HNRNPM	-1.15	2.10E-02	heterogeneous nuclear ribonucleoprotein M
23607	CD2AP	-1.15	2.74E-02	CD2-associated protein
309	ANXA6	-1.15	3.69E-02	annexin A6
2244	FGB	-1.15	3.69E-02	fibrinogen beta chain
1431	CS	-1.15	4.02E-02	citrate synthase
715	C1R	-1.14	2.63E-02	complement component 1, r subcomponent
3263	HPX	-1.14	1.62E-02	hemopexin
1389	CREBL2	-1.14	3.42E-02	cAMP responsive element binding protein-like 2
51585	PCF11	-1.14	4.89E-02	PCF11 cleavage and polyadenylation factor subunit
10096	ACTR3	-1.13	3.46E-02	ARP3 actin-related protein 3 homolog (yeast)
6314	ATXN7	-1.12	3.47E-02	ataxin 7
805	CALM2	-1.12	3.33E-02	calmodulin 2 (phosphorylase kinase, delta)
8553	BHLHE40	-1.12	4.29E-02	basic helix-loop-helix family, member e40
3315	HSPB1	-1.12	4.45E-02	heat shock 27kDa protein 1
7533	YWHAH	-1.12	4.62E-02	tyrosine 3-monooxygenase/tryptophan 5-monooxygenase activation protein, eta
975	CD81	-1.12	2.43E-02	CD81 molecule
